# Enabling Detailed, Biophysics-Based Skeletal Muscle Models on HPC Systems

**DOI:** 10.3389/fphys.2018.00816

**Published:** 2018-07-12

**Authors:** Chris P. Bradley, Nehzat Emamy, Thomas Ertl, Dominik Göddeke, Andreas Hessenthaler, Thomas Klotz, Aaron Krämer, Michael Krone, Benjamin Maier, Miriam Mehl, Tobias Rau, Oliver Röhrle

**Affiliations:** ^1^Auckland Bioengineering Institute, University of Auckland, Auckland, New Zealand; ^2^Institute for Parallel and Distributed Systems, University of Stuttgart, Stuttgart, Germany; ^3^Stuttgart Centre for Simulation Sciences, University of Stuttgart, Stuttgart, Germany; ^4^Visualization Research Center of the University of Stuttgart, University of Stuttgart, Stuttgart, Germany; ^5^Institute for Applied Analysis and Numerical Simulation, University of Stuttgart, Stuttgart, Germany; ^6^SimTech Research Group on Continuum Biomechanics and Mechanobiology, Institute of Applied Mechanics (CE), University of Stuttgart, Stuttgart, Germany

**Keywords:** skeletal muscle mechanics, biophysical modeling, multi-scale modeling, scalability, high-performance computing, numerical efficiency, visualization

## Abstract

Realistic simulations of detailed, biophysics-based, multi-scale models often require very high resolution and, thus, large-scale compute facilities. Existing simulation environments, especially for biomedical applications, are typically designed to allow for high flexibility and generality in model development. Flexibility and model development, however, are often a limiting factor for large-scale simulations. Therefore, new models are typically tested and run on small-scale compute facilities. By using a detailed biophysics-based, chemo-electromechanical skeletal muscle model and the international open-source software library OpenCMISS as an example, we present an approach to upgrade an existing muscle simulation framework from a moderately parallel version toward a massively parallel one that scales both in terms of problem size and in terms of the number of parallel processes. For this purpose, we investigate different modeling, algorithmic and implementational aspects. We present improvements addressing both numerical and parallel scalability. In addition, our approach includes a novel visualization environment which is based on the MegaMol framework and is capable of handling large amounts of simulated data. We present the results of a number of scaling studies at the Tier-1 supercomputer HazelHen at the High Performance Computing Center Stuttgart (HLRS). We improve the overall runtime by a factor of up to 2.6 and achieve good scalability on up to 768 cores.

## 1. Introduction

Even “simple” tasks like grabbing an object involve highly coordinated actions of our musculoskeletal system. At the core of such coordinated movements are voluntary contractions of skeletal muscles. Understanding the underlying mechanism of recruitment and muscle force generation is a challenging task and subject to much research (e.g., Kandel et al., 2000; MacIntosh et al., 2006). One of the few non-invasive and clinically available diagnostic tools to obtain insights into the functioning (or disfunctioning) of the neuromuscular system are electromyographic (EMG) recordings, i. e., measuring the activation-induced, resulting potentials on the skin surface (e.g., Merletti and Parker, 2004). Conclusions on the neuromuscular system are often drawn from results obtained through signal processing, although such signal processing techniques typically ignore the underlying muscular structure. Further limitations of (surface) EMG measurements are, for example, that they only capture activity from muscle parts close to the surface. This leads to difficulties in identifying, for example, cross-talk (e.g., Farina et al., 2005). Moreover, an EMG often only records weak signals due to layers of adipose tissue, and, in some cases, is restricted to isometric contractions. Hence, to obtain more holistic insights into the neuromuscular system, computational models can be employed (for a review see e.g., Mesin, 2013). Such models need to capture much of the electro-mechanical properties of skeletal muscle tissue and the interaction between neural recruitment and muscular contraction.

The contractile behavior of skeletal muscle tissue has been extensively modeled using lumped-parameter models such as Hill-type skeletal muscle models (e.g., Zajac, 1989), continuum-mechanical skeletal muscle models (e.g., Johansson et al., 2000; Blemker et al., 2005; Röhrle and Pullan, 2007; Böl and Reese, 2008), or multi-scale, chemo-electromechanical skeletal muscle models (e.g., Röhrle et al., 2008, 2012; Hernández-Gascón et al., 2013; Heidlauf and Röhrle, 2013). To predict the resulting EMG of a particular stimulation, there exist analytical models (e.g., Dimitrov and Dimitrova, 1998; Farina and Merletti, 2001; Mesin and Farina, 2006) with short compute times, or numerical approaches (e.g., Lowery et al., 2002; Mesin and Farina, 2006; Mordhorst et al., 2015, 2017). For realistic muscle geometries, however, numerical methods are almost unavoidable. The chemo-electromechanical models as proposed by Röhrle et al. (2012), Heidlauf and Röhrle (2013, 2014), or Heidlauf et al. (2016) are particularly well-suited to incorporate many structural and functional features of skeletal muscles. They embed one-dimensional computational muscle fibers within a three-dimensional skeletal muscle model and associate them with a particular motor unit. Moreover, those models can be directly linked to motor neuron models either phenomenologically (e.g., Heckman and Binder, 1991; Fuglevand et al., 1993) or biophysically (e.g., Cisi and Kohn, 2008; Negro and Farina, 2011) to further investigate the relationship between neural and mechanical behavior. The desired degree of detail and complexity within these models requires the coupling of different physical phenomena on different temporal and spatial scales, e.g., models describing the mechanical or electrical state of the muscle tissue on the organ scale and the bio-chemical processes on the cellular scale (cf. section 2.1).

Being able to take into account all these different processes on different scales requires a flexible multi-scale, multi-physics computational framework and significant compute power. The availability of computational resources restricts the number of individual muscle fibers that can be considered within a skeletal muscle. The chemo-electromechanical models as implemented within the international open-source libraries OpenCMISS (e.g., Bradley et al., 2011; Heidlauf and Röhrle, 2013; Mordhorst et al., 2015) allow general muscle geometries with about 1,000 embedded computational muscle fibers. As most skeletal muscles, however, have significantly more fibers (ranging from several thousands to more than a million McCallum, 1898; Feinstein et al., 1955), the embedded muscle fibers geometrically represent only a selection from the actual muscle fibers located in its geometrical vicinity. While simulations with 1,000 fibers and less can potentially provide some insights into the neuromuscular system, some effects, such as the motor unit recruitment over the full range of motor units and muscle fibers and their implication on the resulting EMG, can not be estimated unless a detailed and realistic model with a realistic number of muscle fibers is simulated. This full model allows us to estimate the accuracy of “reduced” models by comparing them to the output of the detailed full “benchmark” model. Unless such comparisons are carried out it is hard to make predictions on how additional details such as, for example, more fibers or functional units (motor units) affect the overall outcome—both in terms of muscle force generation and in terms of computed EMG signals.

Highly optimized and highly parallel software exist in the community for biomechanical applications, e.g., for chemo-electromechanical heart models (Xia et al., 2012; Lafortune et al., 2012; Gurev et al., 2015; Colli Franzone et al., 2015). Skeletal muscle tissue and cardiac muscle tissue share many similarities with respect to the underlying microstructure. Therefore similar simulation techniques can be utilized both for heart models and skeletal muscle models. However, significant differences exist with respect to recruitment and action potential propagation between cardiac and skeletal muscle tissue. Whilst there is a homogeneous and continuous spreading of the action potential across a three-dimensional myocardium, the behavior of skeletal muscle exhibits highly heterogeneous recruitment and action potential propagation—essentially each muscle fiber can be recruited independently leading to complex potential fields. Moreover, there exist feedback mechanisms, e.g., afferent feedback, that directly modulate recruitment. To simulate such complex physiological behavior, one requires flexible computing frameworks and a careful analysis of different parallelization strategies for specific applications like skeletal muscle recruitment.

Most multi-purpose computational frameworks for biomedical applications such as OpenCMISS, for example, are developed to provide flexibility using parallel simulation environments, but are typically not designed for highly parallel simulations on Tier-1 supercomputers. This flexibility is achieved through standards like CellML (e.g., Lloyd et al., 2004) and FieldML (e.g., Christie et al., 2009). The respective frameworks are utilized to enhance existing multi-physics models for a wide range of (bioengineering) applications. Most computational frameworks are designed to be run by biomedical researchers on small-sized compute clusters. While they typically can be compiled on large-scale HPC compute clusters such as HazelHen at the HLRS in Stuttgart, they often are not capable of exploiting the full potential of the hardware for a number of reasons. Moreover, simulation run time is typically considered less important than model complexity and output. Hence, typical simulations of biomedical applications are not necessarily optimized for numerical efficiency, parallel scalability, the exploitation of novel algorithms, or file I/O. In this paper, we demonstrate how one can exploit analysis tools, suitable numerical techniques, and coupling strategies to obtain an efficient chemo-electro-mechanical skeletal muscle model that is suitable to be run on a large-scale HPC infrastructure. The model is thus capable of running with a sufficient resolution and number of muscle fibers to provide the required high-resolution details. Once large-scale simulations of biomedical applications have been solved with a high degree of detail, most specialized visualization tools such as OpenCMISS-Zinc can no longer handle the large amount of simulation data. Dedicated visualization tools for large-scale visualizations are required. In this work, the MegaMol framework (Grottel et al., 2015) has been adapted to visualize the different biophysical simulation parameters and the resulting EMG.

## 2. Model and methods

### 2.1. The multi-scale skeletal muscle model

Before outlining our the model in its full detail, we first provide a brief overview on some anatomical and physiological characteristics of skeletal muscles that are relevant. From an anatomical point of view, skeletal muscles are a hierarchical system. Starting from its basic unit, the so-called sarcomere, several sarcomeres arranged in-series and in-parallel constitute a cylindrically shaped myofibril. Several myofibrils arranged in-parallel make up a skeletal muscle fiber and multiple muscle fibers form a fascicle. All the fascicles together constitute an entire muscle and these fascicles are connected together through the extracellular matrix (ECM). From a physiological point of view, several fibers are controlled by a single lower motor neuron through nervous axons. The entire unit consisting of the lower motor neuron, the axons and the respective fibers that are innervated by the axons, is referred to as a motor unit. The motor unit is the smallest unit within a skeletal muscle that can voluntarily contract. The lower motor neuron sends rate-coded impulses called action potentials (AP) to all fibers belonging to the same motor unit (neural stimulation). Moreover, motor units are activated in an orderly fashion, starting from the smallest, up to the largest (recruitment size principle). After a motor neuron stimulates a muscle fiber at the neuromuscular junction, an AP is triggered and propagates along the muscle fiber, resulting in a local activity (contraction). For more comprehensive insights into muscle physiology and anatomy, we refer to the book of MacIntosh et al. (2006).

As the focus of this research is on enabling the simulation of biophysically detailed skeletal models on HPC architectures, this section provides an overview of the multi-scale modeling framework of our chemo-electromechanical skeletal muscle model that is based on the work by Röhrle et al. (2012), Heidlauf and Röhrle (2013, 2014), and Heidlauf et al. (2016). These models can account for the main mechanical and electro-physiological properties of skeletal muscle tissue, including a realistic activation process and resulting force generation. These results are realized by linking multiple sub-models, describing different physical phenomena on different length and time scales. To reduce the computational costs, the different sub-models are simulated using different discretizations, i. e., spatial resolution and time-step size. Data are exchanged between the sub-models using homogenization and interpolation techniques. The link to neuromuscular recruitment, i.e., an entire neuromuscular model, is modeled using predefined stimulation trains for the fibers associated with individual motor units. This recruitment assumption can be replaced without any modifications with a biophysical motor neuron model (e.g., Cisi and Kohn, 2008; Negro and Farina, 2011).

#### 2.1.1. The 3D continuum-mechanical muscle model

The physiological working range of skeletal muscles includes large deformations. Therefore, we use a continuum mechanical modeling approach that is based on the theory of finite elasticity to simulate the macroscopic deformations and stresses in the muscle tissue. In continuum mechanics, the placement function χ describes the motion of a material point, i. e., it assigns every material point with position ***X*** in the reference (non-deformed) domain $\Omega_{0}\subset {\mathbb{R}}^{3}$ at a time *t*_0_ to a position ***x*** = χ(**X**, *t*) in the actual (deformed) domain $\Omega_{t}\subset {\mathbb{R}}^{3}$ at time *t*. The deformation of the body at a material point can bedescribed by the deformation gradient tensor $F:\text{​​ }=\frac{\partial \chi }{\partial X}=\frac{\partial x}{\partial X},$ which is defined as the partial derivative of the placement function χ with respect to the reference configuration. The local displacement is defined by the vector ***u*** = ***x*** − ***X***.

The governing equation of the continuum mechanical model is the balance of linear momentum. Under the assumption of no acceleration (i.e., inertia forces vanish) and neglecting body forces, the balance of linear momentum in its local form can be written as

(1) $$\text{div }P\;=\;0\text{ in }\Omega_{t}\text{ for all }t,$$

where div(·) denotes the divergence operator and ***P*** is the first Piola-Kirchhoff stress-tensor. To solve the balance of linear momentum, one needs to define a constitutive equation that relates ***P*** to deformation. The constitutive equation describes the overall mechanical behavior of the muscle and can be divided into a passive and an active component. The latter represents the muscle's ability to contract and produce forces. In this work, we assume a superposition of the active and passive behavior, i. e., an additive split of ***P***.

Passive skeletal muscle tissue is assumed to be hyperelastic and transversely isotropic. Consequently, the passive part to the first Piola-Kirchhoff stress tensor $P_{\text{passive}}(F,M)$ depends on the deformation gradient tensor ***F*** and a structure tensor $M=a_{0}⊗a_{0}$ , which is defined by the muscle fiber direction ***a***_0_. The isotropic part of the passive stress-tensor assumes a Mooney-Rivlin material. It is enhanced by an additive anisotropic contribution accounting for the specific material properties in the muscle fiber direction ***a***_0_.

The active force is generated on a microscopic scale, i.e., within a half-sarcomere (the smallest functional unit of a muscle) consisting of thin actin and thick myosin filaments. Based on geometrical considerations of the half-sarcomere structure, it is known that the active muscle force depends on the actual half-sarcomere length *l*_hs_ (force-length relation) (Gordon et al., 1966). When a half-sarcomere is activated by calcium as a secondary messenger, actin and myosin filaments can form cross-bridges and produce forces (cross-bridge cycling). The active force state of the microscopic half-sarcomere is summarized in an activation parameter γ that enters the macroscopic constitutive equation. Furthermore, we assume that the active stress contribution acts only along the fiber direction ***a***_0_. When considering only isometric or slow contractions, the active stress tensor $P_{\text{active}}(F,M,\gamma )$can be defined as a function of the lumped activation parameter γ, the deformation gradient tensor ***F***, and the structure tensor M. An additional force-length relationship needs to be included within ***P***_active_.

Finally, we assume skeletal muscle tissue to be incompressible, which implies the incompressibility constraint det***F*** = 1. The resulting first Piola-Kirchhoff stress tensor reads

(2) $$P(F,M,\gamma )\;=\;P_{\text{passive}}(F,M)+P_{\text{active}}(F,M,\gamma )-pF^{-T}\;,$$

where *p* is the hydrostatic pressure, which enters the equation as a Lagrange multiplier enforcing the incompressibility constraint. The material parameters of the continuum-mechanical skeletal muscles are fitted to experimental data (Hawkins and Bey, 1994), and can be found in Heidlauf and Röhrle (2014).

#### 2.1.2. The 1D model for action potential propagation

The electrical activity of skeletal muscles resulting from the local activity of all muscle fibers can be analyzed by measuring the extracellular potential. The bidomain-model is a framework widely used in continuum mechanics to simulate the electrical activity of living tissues (Pullan et al., 2005). It is based on the assumption that the intracellular and extracellular spaces homogeneously occupy the same domain. The intracellular and extracellular spaces are electrically coupled by an electrical current *I*_m_ flowing across the cell membrane, i. e.,

$$\begin{array}{l} \begin{array}{c} -\mathrm{div}\;q_{\text{i}}=\mathrm{div}\;q_{\text{e}}=A_{\text{m}}I_{\text{m}}, \end{array} \end{array}$$

where ***q***_i_ and ***q***_e_ denote the current density in the intracellular and extracellular space, respectively, and *A*_m_ is the fiber's surface to volume ratio. The muscle fiber membrane is nearly impermeable for ions and serves as a capacitor. However, ions can be transported through the membrane by ion channels and active ion pumps. This process can be mathematically described by the biophysically motivated modeling approach proposed by Hodgkin and Huxley (1952) which leads to the constitutive equation

(3) $$\begin{array}{l} I_{\text{m}}\;=\;C_{\text{m}}\frac{\partial V_{\text{m}}}{\partial t}+I_{\text{ion}}\left(y,V_{\text{m}},I_{\text{stim}}\right)\;, \end{array}$$

where *V*_m_ is the transmembrane potential, *C*_m_ is the capacitance of the muscle fiber membrane (sarcolemma) and *I*_ion_ is the transmembrane-potential-dependent ionic current flowing through the ion-channels and -pumps. Further state variables are summarized in ***y***, e. g., the states of different ion channels. *I*_stim_ is an externally applied stimulation current, e. g., due to a stimulus from the nervous system. Assuming that the intracellular space and extracellular space show the same anisotropy, which is the case for 1D problems, the bidomain equations can be reduced to the monodomain equation. We thus use the one-dimensional monodomain equation in the domain Γ_*t*_ ⊂ ℝ:

(4) $$\frac{\partial V_{\text{m}}}{\partial t}=\frac{1}{A_{\text{m}}C_{\text{m}}}\left(\frac{\partial }{\partial x}(\sigma_{\text{eff}}\frac{\partial V_{\text{m}}}{\partial x})-A_{\text{m}}I_{\text{ion}}\left(y,V_{\text{m}},I_{\text{stim}}\right)\right)\text{ in }\Gamma_{t}.$$

Here, *x* denotes the spatial coordinate along a one-dimensional line, i.e., the fiber, and σ_eff_ is the effective conductivity.

#### 2.1.3. The 0D sub-cellular muscle model

The model proposed by Shorten et al. (2007) provides a basis to compute the lumped activation parameter γ, which is the link to the 3D continuum-mechanical muscle model. Its evolution model is steered by the ionic current *I*_ion_ of the 1D model. In more detail, the 0D sub-cellular muscle model contains a detailed biophysical description of the sub-cellular excitation-contraction coupling pathway. Specifically, it models the depolarization of the membrane potential in response to stimulation, the release of calcium from the sarcoplasmic reticulum (SR) which serves as a second messenger, and cross-bridge (XB) cycling. To do so, the Shorten model couples three sub-cellular models: A Hodgkin-Huxley-type model is utilized to simulate the electrical potentials and ion currents through the muscle-fiber membrane and the membrane of the T-tubule system. For calcium dynamics, a model of the SR membrane ryanodine receptor (RyR) channels (Ríos et al., 1993) is coupled to the electrical potential across the T-tubule membrane and models the release of calcium from the SR. Additionally, the calcium-dynamics model describes diffusion of calcium in the muscle cell, active calcium transport through the SR membrane via the SERCA pump (sarco/endoplasmic reticulum Ca^2+^-ATPase), binding of calcium to buffer molecules (e. g. , parvalbumin or ATP), and binding of calcium to troponin enabling the formation of cross-bridges. The active force generation is simulated by solving a simplified Huxley-type model (Razumova et al., 1999), which is the basis for calculating the activation parameter γ.

All incorporated sub-cellular processes are modeled with a set of coupled ordinary differential equations (ODEs)

(5) $$\begin{array}{l} \frac{\partial y}{\partial t}\;=G_{y}\left(y,V_{\text{m}},I_{\text{stim}}\right), \end{array}$$

where *G*_***y***_ summarizes the right-hand-side of all the ODEs associated with the state variables ***y*** which number, in the case of the Shorten et al. model, more than 50.

The final activation parameter γ is computed from the state variable vector ***y*** and the length and contraction velocity of the half-sarcomere, *l*_hs_ and ${\dot{l}}_{\text{hs}}$. For isometric or very slow contractions, the contraction velocity can be neglected. Hence, following Razumova et al. (1999) and Heidlauf and Röhrle (2014), the activation parameter is calculated as

(6) $$\begin{array}{l} \gamma \left(y,l_{\text{hs}}\right)=f_{\text{f}\text{-l}}\left(l_{\text{hs}}\right)\frac{A_{2}-A_{2}^{\text{min}}}{A_{2}^{\text{max}}-A_{2}^{\text{min}}}\;. \end{array}$$

Here, the function *f*_f-l_(*l*_hs_) is the force-length relation for a cat skeletal muscle by Rassier et al. (1999), A_2_ ∈ ***y*** is the concentration of post power-stroke cross-bridges, $A_{2}^{\text{max}}$ is the concentration of post power-stroke cross-bridges for a tetanic contraction (100 Hz stimulation after 500 ms stimulation) and $A_{2}^{\text{min}}$ is an offset parameter denoting the concentration of post power-stroke cross-bridges in the resting state.

#### 2.1.4. Summary of the full model

In summary, the chemo-electromechanical behavior of a skeletal muscle is described by the following coupled equations:

(7a) $$0\;=\text{ div }P(F,M,\gamma (y,l_{\text{hs}}))\text{ in }\Omega_{t}forall\;t,$$

(7b) $$\begin{array}{l} \frac{\partial V_{\text{m}}}{\partial t}\;=\;\frac{1}{A_{\text{m}}C_{\text{m}}}(\frac{\partial }{\partial x}(\sigma_{\text{eff}}\frac{\partial V_{\text{m}}}{\partial x})\\ \;-A_{\text{m}}I_{\text{ion}}(y,V_{\text{m}},I_{\text{stim}}))\text{ on all fibers }\Gamma_{t}, \end{array}$$

(7c) $$\;\frac{\partial y}{\partial t}\;=\;G_{y}\left(y,V_{\text{m}},I_{\text{stim}}\right)\text{ at all sarcomere positions}.$$

Realistic material parameters and muscle fiber directions, appropriate boundary and initial condition (i.e., Dirichlet boundary conditions for the three-dimensional, continuum-mechanical model to describe the displacement of a tendon and, hence, of the skeletal muscle tissue, as a result of motion, or the stimulus train, *I*_stim_(*t*)) for all fibers, need to be chosen (cf. section 3.1 for a particular example).

### 2.2. Numerical methods

To enable multi-scale skeletal muscle models, e.g., such as the ones described in section 2.1, to run efficiently and scalably on (large-scale) clusters, we first present the numerical methods as implemented in Heidlauf and Röhrle (2013) (section 2.2.1) followed by algorithmic optimizations aiming to achieve efficient and scalable code (section 2.2.2). To distinguish between the implementation of Heidlauf and Röhrle (2013) and the new optimized implementation, we denote the former as the baseline implementation.

#### 2.2.1. Discretization and solvers

##### 2.2.1.1. Spatial discretization

**Figure 1:**
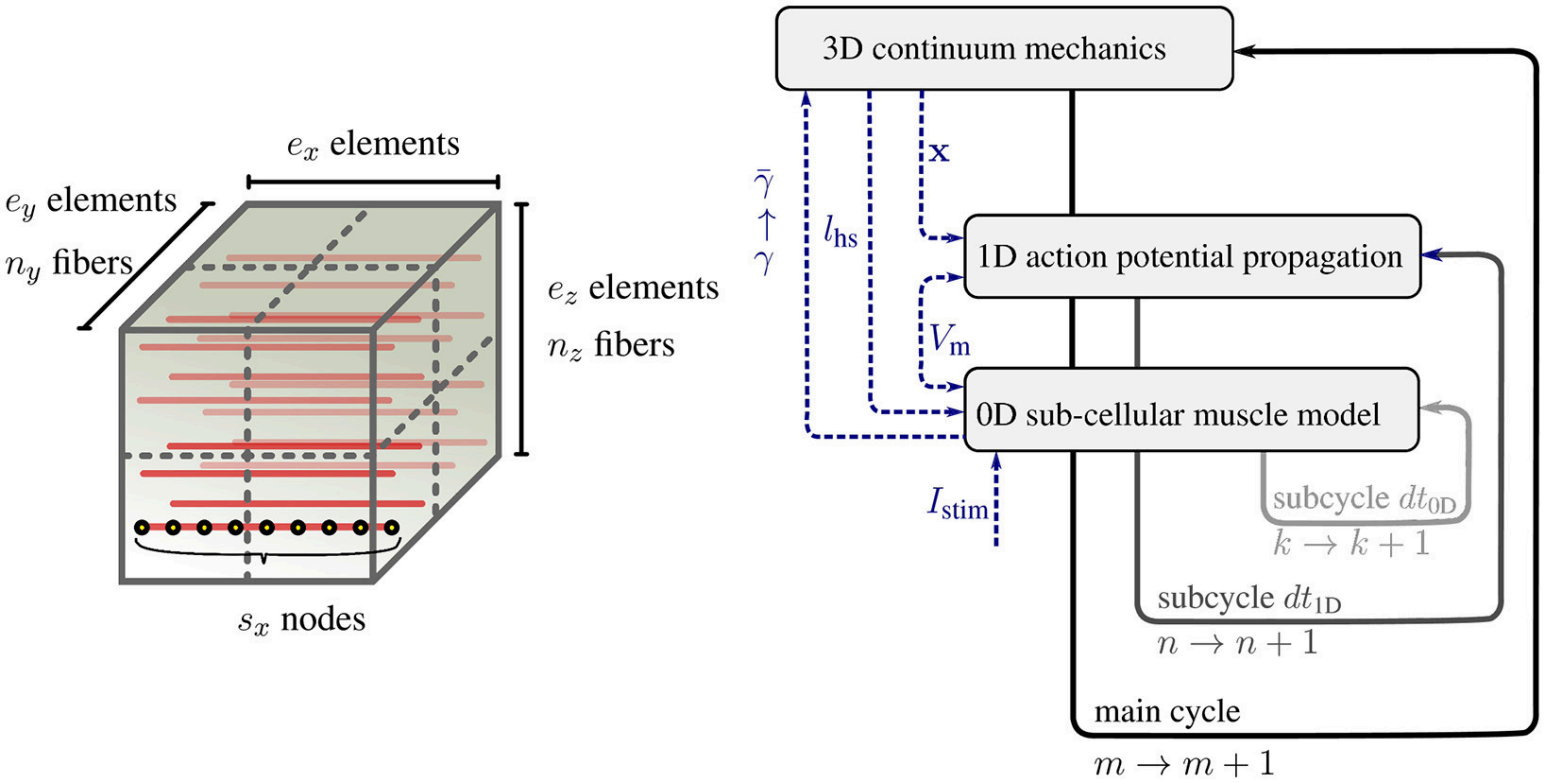
**(Left)** Schematic view of a 3D muscle domain that contains a given number of *n*_*x*_ × *n*_*y*_ muscle fibers per 3D partition, *e*_*x*_ × *e*_*y*_ × *e*_*z*_ finite elements for the 3D model (7a), and *s*_*x*_ nodes per fiber for (7b) and (7c). **(Right)** Schematic view of the multi-scale time stepping scheme based on a Godunov splitting of the monodomain equation.

The sub-models of the multi-scale skeletal muscle model have significantly different characteristic time and length scales. To solve the overall model efficiently, different discretization techniques and resolutions are required for the sub-models. In Heidlauf and Röhrle (2013), as in this work, the continuum-mechanics model is solved via the finite element method using Taylor-Hood elements (i. e., a mixed formulation of tri-quadratic and tri-linear Lagrange basis functions to approximate the displacements and the hydrostatic pressure respectively). The one-dimensional muscle fibers are represented by embedded, one-dimensional finite element meshes with linear Lagrange basis functions. Figure 1 (left) shows the embedding of *n*_*y*_ × *n*_*z*_ discretised 1D fibers within the 3D muscle domain Ω_0_ discretised with *e*_*x*_ × *e*_*y*_ × *e*_*z*_ tri-quadratic finite elements, where *e*_*x*_, *e*_*y*_, and *e*_*z*_ are the number of elements in the *x*, *y*, and *z* direction respectively. Each node of the 1D fiber mesh serves as sarcomere position where one instance of the sub-cellular model is calculated.

The different discretizations of the coupled multi-physics problem require data to be transfered between the different spatial discretizations. Within our model, the transfer of information from the microscopic scale to the macroscopic scale is realized via the activation parameter γ. The microscopic sarcomere forces γ provided by the monodomain model are projected to the macroscopic three-dimensional continuum-mechanics model ($\gamma \to \overset{-}{\gamma }$). This homogenization is performed for all Gauss points in the 3D model by averaging the γ values of all monodomain model nodes nearest to the respective Gauss point. Similarly, the node positions of the one-dimensional computational muscle fibers are updated from the actual displacements ***u*** of the three-dimensional, continuum-mechanics model by interpolating the node positions via the basis functions of the three-dimensional model. Based on this step, the microscopic half-sarcomere lengths *l*_hs_(***x***) can be calculated.

##### 2.2.1.2. Time discretization

To compute an approximate solution for Equation (7), the different characteristic time scales of the 3D, 1D and 0D problems can be exploited. The action potential propagates faster than the muscle deformation, and the sub-cellular processes evolve considerably faster than the diffusive action potential propagation. From a computational point of view, it is desirable to have common global time steps. To achieve this, we choose *dt*_3D_/N = *dt*_1D_ = K·*dt*_0D_ with N, K ∈ ℕ. Then, each discrete time is uniquely defined as *t*_*m, n, k*_: = *m* · *dt*_3D_ + *n*·*dt*_1D_ + *k*·*dt*_0D_, with M ∈ ℕ, *n* = 0, .., *N* − 1 and *k* = 0, .., *K* − 1. Moreover, state values associated with time *t*_*m, n, k*_ are denoted with the superscript (·)^*m, n, k*^. Employing different time steps requires a time splitting scheme. The baseline implementation in Heidlauf and Röhrle (2013) uses a first-order accurate Godunov splitting scheme, for which one time-step of the three-dimensional equation including all sub-steps for the one-dimensional monodomain equation is given by:

1. For *n* = 0, …, *N* − 1 do
  - For *k* = 0, …, *K* − 1 perform explicit Euler steps for Equation (7c) and the 0D portion of Equation (7b).
  - Set $V_{\text{m}}^{m,n,0}:\;=V_{\text{m}}^{m,n,K}$ and ***y***^*m, n*+1, 0^: = ***y***^*m, n, K*^.
  - Perform one implicit Euler step for the 1D portion of Equation (7b) to compute $V_{\text{m}}^{m,n+1,0}$.
2. Set $V_{\text{m}}^{m+1,0,0}:\;=V_{\text{m}}^{m,N,0}$ and ***y***^*m*+1, 0, 0^: = ***y***^*m, N*, 0^.
3. Calculate $\gamma \left(y^{m+1,0,0},l_{\text{hs}}^{m,0,0}\right)$ and compute the homogenized values $\overset{-}{\gamma }$ at the Gauss points of the 3D finite element mesh.
4. Calculate the activation parameter $\gamma \left(y^{m+1},l_{hs}^{m}\right)$.
5. Solve Equation (7a).
6. Interpolate the actual configuration ***x***^*m*+1,0,0^ to the fibers' nodes for computing the local half-sacromere length $l_{\text{hs}}^{m+1,0,0}$.

Figure 1 (right) schematically depicts this algorithm.

##### 2.2.1.3. Linear solvers

The coupled time stepping algorithm described above contains two large systems of equations that need to be solved. The first one results from the 3D elasticity problem (7a) and the second one stems from an implicit time integration of the linear 1D diffusion problem of the fiber (7b). In Heidlauf and Röhrle (2013) the linear systems are obtained by applying Newton's method to the 3D and 1D problems and are solved using GMRES (Saad and Schultz, 1986) as implemented within the PETSc library (Balay et al., 1997, 2015).

#### 2.2.2. Algorithmic optimizations

While section 2.2.1 describes the implementation as in Heidlauf and Röhrle (2013), in the following paragraphs we propose some algorithmic optimizations to improve numerical efficiency.

##### 2.2.2.1. Spatial discretization

We optimize the interpolation and homogenization routines, and leave the spatial discretization as described in section 2.2.1 unchanged in this work: interpolation and homogenization steps involve the transfer of information between values at Gauss points of the 3D elements to nodes of the 1D fibers. To allow for a general domain decomposition later on, a mapping between the respective 3D and 1D finite elements is necessary. In Heidlauf and Röhrle (2013), the homogenization was achieved using a naive search over all locally stored fibers. This search was performed for each 3D element. We replace this approach, which exhibits quadratic complexity (in terms of the number of involved elements), with a calculation of linear complexity. This is achieved by calculating – in constant time – the indices of the 1D elements that are located inside a 3D element.

##### 2.2.2.2. Second-order time stepping

To reduce computational cost, we replace the first-order Godunov splitting with a second-order Strang splitting as proposed by, e.g., Qu and Garfinkel (1999). A higher order means that we advance from an O(dt) approach to an $O\left(dt^{2}\right)$ for a given steplength *dt* in time. Second-order time-stepping schemes reduce the discretization error much faster with a decreasing time step size *dt* and thus, the required accuracy might be achieved using larger time steps. Along with the change of the splitting approach, we replace the explicit Euler method for Equation (7c) and the 0D portion of Equation (7b) with the method of Heun and employ an implicit Crank-Nicolson method for the diffusion part of Equation (7b). In contrast to the simpler Godunov splitting, Strang splitting uses three sub-steps per time step: a first step with length *dt*_1D_/2 for the 0D part, a second step with length *dt*_1D_ for the diffusion, and a third step with length *dt*_1D_/2 again for the 0D part. The modified algorithm at time *t*_*m*, 0, 0_ is given by:

1. For *n* = 0, …, *N* − 1 do
  - For *k* = 0, …, *K*/2 − 1 perform explicit Heun steps for Equation (7c) and the 0D portion of Equation (7b).
  - Set $V_{\text{m}}^{m,n,0}:\;=V_{\text{m}}^{m,n,K/2}$.
  - Perform one implicit Crank-Nicolson step for the 1D portion of Equation (7b).
  - Set $V_{\text{m}}^{m,n,K/2}:\;=V_{\text{m}}^{m,n+1,0}$.
  - For *k* = *K*/2, …, *K* − 1 perform explicit Heun steps for Equation (7c) and the 0D portion of Equation (7b).
  - Set $V_{\text{m}}^{m,n+1,0}:\;=V_{\text{m}}^{m,n,K}$ and ***y***^*m, n*+1, 0^: = ***y***^*m, n, K*^.
2. Set $V_{\text{m}}^{m+1,0,0}:\;=V_{\text{m}}^{m,N,0}$ and ***y***^*m*+1, 0, 0^: = ***y***^*m, N*, 0^.
3. Calculate $\gamma \left(y^{m+1,0,0},l_{\text{hs}}^{m,0,0}\right)$ and compute the homogenized values $\overset{-}{\gamma }$ at the Gauss points of the 3D finite element mesh.
4. Solve Equation (7a).
5. Interpolate the displacements ***u***^*m*+1, 0, 0^ to the fibers' nodes for computing the local half-sacromere length $l_{\text{hs}}^{m+1,0,0}$.

The explicit Heun step in 1.a. and 1.d. (see above) is given by:

(8a) $${\left[\text{​}\begin{array}{c} y\\ V_{\text{m}} \end{array}\text{​}\right]}^{\text{pre}}\text{​​​​​}=\text{​​}{\left[\text{​}\begin{array}{c} y\\ V_{\text{m}} \end{array}\text{​}\right]}^{m,n,k}\text{​​}+dt_{\text{0D}}\text{​}\left[\text{​}\begin{array}{l} G_{y}(y^{m,n,k},V_{\text{m}}^{m,n,k},I_{\text{stim}})\\ -\frac{1}{C_{\text{m}}}I_{\text{ion}}(y^{m,n,k},V_{\text{m}}^{m,n,k},I_{\text{stim}}) \end{array}\right],$$

(8b) $$\begin{array}{l} {\left[\text{​}\begin{array}{c} y\\ V_{\text{m}} \end{array}\text{​}\right]}^{m,n,k+1}\text{​​}=\text{ ​​}{\left[\text{​}\begin{array}{c} y\\ V_{\text{m}} \end{array}\text{​}\right]}^{m,n,k}\\ \text{​​​​​​​​​​​​​​​​​​​​ }+\frac{dt_{\text{0D}}}{2}\left[\text{​}\begin{array}{c} G_{y}(y^{m,n,k},V_{\text{m}}^{m,n,k},I_{\text{stim}})\\ +G_{y}(y^{\text{pre}},V_{\text{m}}^{\text{pre}},I_{\text{stim}})\\ -\frac{1}{C_{\text{m}}}(I_{\text{ion}}(y^{m,n,k},V_{\text{m}}^{m,n,k},I_{\text{stim}})\\ +I_{\text{ion}}(y^{\text{pre}},V_{\text{m}}^{\text{pre}},I_{\text{stim}})) \end{array}\text{​}\right] \end{array}$$

In 1.b., we solve the system resulting from the Crank-Nicolson time discretization of the diffusion part in Equation (7b):

(9) $$\begin{array}{l} V_{\text{m}}^{m,n+1,0}=V_{\text{m}}^{m,n,0}+\frac{dt_{\text{1D}}}{2A_{\text{m}}C_{\text{m}}}(\frac{\partial }{\partial x}\left(\sigma_{\text{eff}}\frac{\partial V_{\text{m}}^{m,n,0}}{\partial x}\right)\\ \;+\frac{\partial }{\partial x}\left(\sigma_{\text{eff}}\frac{\partial V_{\text{m}}^{m,n+1,0}}{\partial x}\right)), \end{array}$$

##### 2.2.2.3. Optimal complexity linear solver

The GMRES solver is a robust choice for general sparse systems of linear equations but it does not exploit the symmetry, positive definiteness and tri-diagonal structure of the 1D diffusion system. For symmetric matrices the conjugate gradient (CG) solver (Hestenes and Stiefel, 1952) is an appropriate iterative solver. For tri-diagonal matrices one could even employ the most simple Thomas algorithm (Thomas, 1949). To maintain flexibility, we currently replace the GMRES solver by a direct solver from the MUMPS library (Amestoy et al., 2001, 2006) that exploits the structure and exhibits optimal complexity for tridiagonal systems.

### 2.3. Domain partitioning and parallelization

For parallelization, the computational domains must be partitioned appropriately. This is particularly challenging for multi-scale problems, as considered in this work, as the parallelization induces communication due to dependencies of local data on data in neighboring partitions. To motivate the discussion below, we briefly outline the main challenges in the scope of this work:

1. Solving for the propagation of *V*_m_, i. e., using an implicit Euler or Crank-Nicolson method (equation 9) to solve the monodomain equation (equation 4), requires communication of data along a single fiber. The resulting communication cost per process is thus linear in the number of fibers that are split in the global 3D partitioning, and whose parts are thus assigned to different partitions.
2. Computing the muscle displacements ***u*** of the 3D model involves all processes. This is a result of using a finite element discretization, which inherently requires peer-to-peer communication between processes which share partition boundaries. These costs are proportional to the surface area of the 3D partitions.
3. Interpolating the muscle displacements ***u*** of the 3D muscle mesh to 1D fiber mesh node positions and calculating *l*_hs_, requires ghost layers at the partition boundaries containing one layer of 3D elements. Note that for the reverse transfer, the accumulation of the activation parameter γ from the 0D model at the Gauss points of the elements in the 3D mesh, i. e., computing $\overset{-}{\gamma }$, does not involve communication since the process is completely local as all 0D points are contained within the respective 3D element and reside on the same process.

#### 2.3.1. Pillar-like domain decomposition

In the baseline implementation by Heidlauf and Röhrle (2013), the domain decomposition for parallel execution was hard-coded for only four processes, following a partitioning ensuring that entire fibers remain within the same partition at all times, which is anatomically motivated. Since all skeletal muscle fibers are, from an electrical point of view, independent of each other, this is also computationally attractive as no quantities in the 0D and 1D sub-models need to be exchanged between fibers. We extend the approach to an arbitrary number of processes, and keep the structure of partitioning the 3D and 1D meshes in the same way, such that quantities in the 3D, 1D and 0D models corresponding to the same spatial location are stored on the same process. This avoids unnecessary inter-process volume-communication between the sub-models.

#### 2.3.2. New spatial domain decomposition

**Figure 2:**
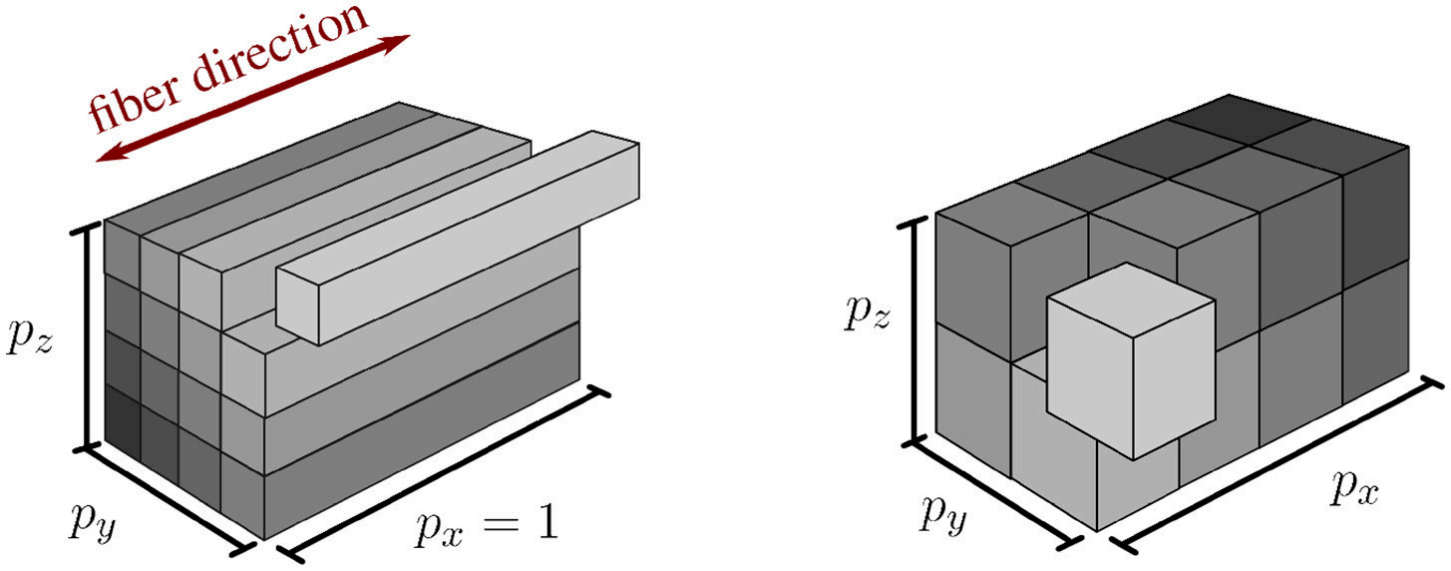
Visualization of pillar-like **(Left)** and cuboid **(Right)** domain decomposition approaches. Both depicted approaches partition the same domain into 16 subdomains with *p*_*x*_, *p*_*y*_, and *p*_*z*_ subdivisions in *x*-, *y*-, and *z*-direction, respectively.

In addition to the extension of the pillar-like domain partitioning, we investigate a second approach with nearly cube-shaped partitions, cf. Figure 2. In contrast to partitioning strategies based on space-filling curves such as Schamberger and Wierum (2005), graph partitioning such as Miller et al. (1993) and Zhou et al. (2010), or problem-specific approaches such as the pillar-shaped partitioning, a cuboid partition has the advantage that the interaction of one cuboid partition with others is guaranteed to be planar and bounded by the maximum number of neighboring partitions, i. e., 3^3^ − 1 = 26. This allows communication with reduced complexity and cost.

However, we cannot completely avoid obtaining sub-domains at the boundary of the computational domain that have less elements than other domains. Given a fixed number of available cores, we thus maximize the number of employed processes by adapting the number of sub-divisions in each axis direction corresponding to a factorization of the total number of processes. By carefully choosing the factorization, we reduce the impact on sub-optimal load-balancing in these ‘nearly cuboid’ partitioning cases. By introducing the additional constraint that each generated partition has to be larger than a specified “atomic” cuboid of elements, we can easily ensure that each process contains only entire fibers (pillar-like partition), a fixed number of fiber subdivisions (cube-like partition), or anything in between.

In summary, based on the communication dependencies 1 and 2 as described at the beginning of this section, we enhance the original pillar-like domain partitioning in two ways: (i) we allow for an arbitrary number of processes instead of a fixed number of four processes and (ii) we introduce a new partitioning concept with nearly cuboid partitions that minimize the partitioning's surface area.

Note, when considering the simulation of realistic muscle geometries that cannot be discretized using rectangular elements, e.g., using unstructured meshes, a domain decomposition into pillar-like or nearly cuboid partitions is generally no longer feasible. The same is true for a skeletal muscle with complex muscle fiber distributions. In such a case, one cannot ensure that fibers are always contained within a single partition when using a pillar-like domain decomposition. However, the strategy to aim for minimal surface domains is always possible as it inherently involves cutting fibers at process boundaries.

Within this work, we assume that it is possible to create nearly optimal cube-shaped partitions.

### 2.4. Visualization of muscle simulations

Performing large scale simulations is only the first step to gain an improved insight into the musculoskeletal system. Visual analysis and interactive exploration of the simulation data gives the opportunity to investigate every facet of large and complex systems. General-purpose visualization tools like ParaView (Ahrens et al., 2005) or VisIt (Childs et al., 2012a) can only provide a first glimpse of such data sets. However, for the above-mentioned in-depth analysis, a tailored visualization tool is necessary. The standard visualization framework within the OpenCMISS software project is OpenCMISS-Zinc. This framework already offers a range of visualization techniques for muscle fiber data, for example, a convex hull calculation to construct a mesh geometry from point cloud data. However, OpenCMISS-Zinc lacks important features that are required to develop efficient visualizations intended to run on HPC systems. These missing features are, for example, a suitable platform for fast visualization prototyping, distributed rendering, or CPU-based visualization. The open-source visualization framework MegaMol (Grottel et al., 2015) fulfills these criteria and offers additional functionality and features that are valuable for this project. Therefore, we use MegaMol as the basis for improved musculoskeletal visualizations. For example, one additional feature is the infrastructure for brushing and linking that allows for developing interactive visual analytics applications. MegaMol also offers a built-in headless mode and a remote control interface, which is crucial for HPC-based in-situ rendering.

In-situ visualization is an alternative approach to traditional *post-hoc* data processing. The key idea is to process and visualize data on the HPC system while the simulation is running. Consequently, writing raw data to disk can be avoided completely. Since our new visualization tool is intended to cope with the visual analysis of large-scale muscle simulations, we require an architecture that allows us to employ this approach in the future. There are three different approaches that are considered as in-situ visualization, identified by Childs et al. (2012b). The first one is known as co-processing, where the visualization tool runs simultaneously with the simulation and accesses the simulations memory for further processing and visualization. In the second approach, the visualization runs on separate nodes and communicates data via a network. This method is known as concurrent-processing. The last possibility, the hybrid technique, directly accesses the simulation's memory and reduces the data for less network load while sending the data to visualization nodes. We are planning to add the first two methods—co-processing and concurrent processing—into our implementation. However, we cannot completely disregard the hybrid technique as we might need to identify the workload of each node and the network traffic of a running large-scale simulation with in-situ visualization first.

Interactive visualization typically uses graphics APIs like OpenGL to employ the GPU for rendering. GPU-accelerated rendering uses polygon rasterization, i. e., large numbers of triangles can be processed and rendered in parallel. All geometric objects that are rendered thus have to be represented by triangle meshes. This visualization approach is, for example, also used by OpenCMISS-Zinc. An alternative rendering approach to GPU-accelerated rasterization is ray tracing. Here, one or more view rays are computed for each pixel. Each ray is tested for intersection with the objects in the scene in order to find out which objects are visible at this pixel. Note that this approach can not only render triangles but also all objects that have a mathematical representation that can be used for computing the ray-object intersection (e. g., spheres or cylinders). Ray tracing is usually computed on the CPU and was traditionally only used for high-quality offline rendering due to its higher computational complexity. The combination of modern hardware and improved algorithms, however, enables interactive ray tracing, even on single desktop workstations.

MegaMol offers GPU rendering (rasterization) and CPU ray tracing via a thin abstraction layer. The GPU rendering uses the OpenGL API, whereas the CPU rendering is based on the ray tracing engine OSPRay (Wald et al., 2017). In particular the CPU-based ray tracing enables image synthesis on any computer, regardless of the availability of dedicated GPUs. This is especially important for HPC clusters, which are typically not equipped with GPUs: Currently, only two of the top-ten HPC systems in the Top500 list GPU systems. Since ray tracing simulates the transport of light, it offers advanced rendering and shading methods (e. g., global illumination and ambient occlusion) that enhance the perception of depth. MegaMol is currently not optimized for HPC usage. However, it provides the necessary basic infrastructure for enabling distributed rendering on an HPC system. Furthermore, MegaMol is already capable of rendering discretized muscle fibers as continuous geometry. The visual quality and scalability obtained by MegaMol using integrated OSPRay ray tracing are discussed in section 3.4.

## 3. Results

Before simulating realistic and complex models on HPC systems, it is essential to first analyse numerical complexity, i. e., scalability in terms of the size of the problem both for the baseline methods described in section 2.2.1 and our optimized methods presented in section 2.2.2. To avoid any geometrical effects stemming from realistic geometries, we perform the analysis on a test example introduced in section 3.1. As the old parallel code used 4 cores, only, in section 3.3 we restrict our analysis of the parallel scalability to the proposed new parallelization strategies.

### 3.1. Test scenario

As a test scenario, we use a generic cubic muscle geometry (1 × 1 × 1cm). The muscle fibers are aligned in parallel to one cube-edge (the *x*-direction). The discretization in space and time is as carried out as described in sections 2.2.1 and 2.2.2. The discretization parameters will be specified for the respective experiments. For the material parameters for the continuum-mechanics model, the effective conductivity σ_eff_, the surface-to-volume ratio *A*_m_, and the membrane capacity *C*_m_, we use exactly the same values as reported in Heidlauf and Röhrle (2014).

To constrain the muscle, Dirichlet boundary conditions (zero displacement) are used to fixate the following faces of the muscle cube: the left and the right faces (faces normal to the *x*-direction), the front face (face normal to the *y*-direction) and the bottom face (face normal to the *z*-direction). Further, no current flows over the boundary of the computational muscle fibers, i. e., zero Neumann boundary conditions are assumed at both muscle fiber ends. As far as the skeletal muscle recruitment is concerned, we consider an isometric single-twitch experiment by stimulating all fibers at their mid-points for *t* ∈ [0, 0.1 ms] with $I_{\text{stim}}\left(t\right)=1200\mu \text{A/}{\text{cm}}^{2}$. For all other *t*, *I*_stim_(*t*) is assumed to be 0.

### 3.2. Numerical investigations

In the following, we present numerical experiments demonstrating, in particular, the increase in efficiency with the new second-order time discretization method. All runtimes are measured in serial, on an Intel® Core™ i5-4590 CPU (3.3 GHz, 32 GB RAM) for Secs. 3.2.1 and 3.2.2, and an Intel® Xeon™ E7-8880 v3 CPU (2.3 GHz, 504 GB RAM) for Secs. 3.2.3, 3.2.4, using the OpenCMISS implementation.

#### 3.2.1. Time discretization for the sub-cellular model

**Figure 3:**
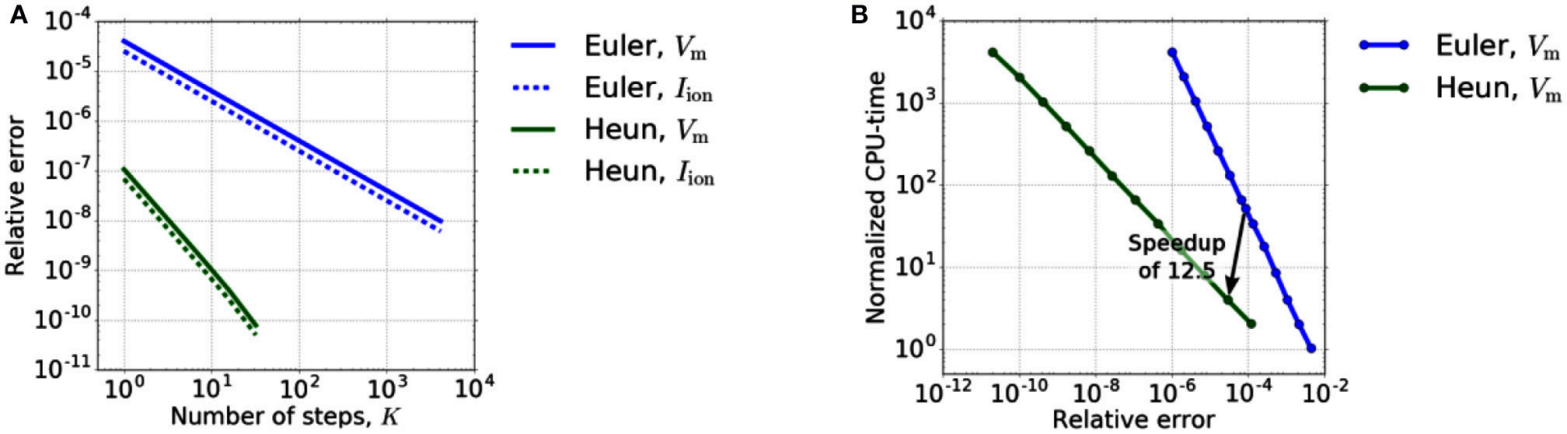
**(A)** Relative error dependency on the number *K* of 0D time steps in [0, *dt*_1D_]. The error of Euler's and Heun's method shows the expected $O\left(K^{-1}\right)$ and $O\left(K^{-2}\right)$ behavior. **(B)** Dependency of the runtime on the required accuracy for explicit Euler and Heun. We varied the time step *dt*_0D_ between 5·2^0^ and 5·2^−12^μs for Euler and between 5·2^0^ and 5·2^−11^μs for Heun.

In a first step, we verify the convergence order of Heun's method experimentally. Therefore, we restrict ourselves to the reaction term, i. e., step 1.a of the Godunov algorithm, but use Heun's method for Equation (7c) and the 0D portion of Equation (7b). The diffusion term is thus completely neglected. We use the test setup as presented in Sect. 3.1. To compare the accuracy of Heun's method with an explicit Euler method, we compare the values of *V*_m_ and *I*_ion_ at a stimulated material point on a muscle fiber while varying the time step size *dt*_0D_. As a reference solution, we use the solution calculated with Heun's method for a very high resolution (*K*: = *dt*_1D_/*dt*_0D_ = 4096). We restrict ourselves to the time interval [0, *dt*_1D_], with *dt*_1D_ = 0.5 μs. To compare the methods in terms of efficiency, we measure the related compute times. Figure 3A depicts the relative error depending on the number *K* of 0D time steps while on the right the necessary CPU-times to reach a certain accuracy for the different solvers are compared.

Figure 3A shows the expected first-order convergence for the explicit Euler method and second-order convergence for Heun's method. From an application point of view, however, efficient computation (“Which accuracy can be achieved in which runtime?”) is more important than the order of convergence. Therefore, in order to reveal the potential of Heun's method in decreasing the runtime for a given required accuracy, we take into account the different computation time per step of the methods. Figure 3B shows that two Heun steps with *dt*_0D_ = 2.5 μs replace 50 forward Euler steps yielding a theoretical speedup of 12.5 for the 0D-solver. At the same time, the error decreases by a factor of approximately 3. All times are normalized with respect to the CPU-time of a single step of the Euler method (*K* = 1).

#### 3.2.2. Time discretization for the muscle fibers

In a second experiment, we verify the convergence order of the Strang splitting scheme, i. e., we couple 0D reaction and 1D diffusion. Again, the same test setup as above is considered except that we use a larger time interval [0, 0.1 ms] and vary the number, N, of 1D time steps. Based on the previous results for the isolated 0D problem, we choose K = 2 for the Strang-splitting scheme and K = 5 for the Godunov-splitting scheme. This ensures a comparable relative error for the 0D sub-problem while saving computational time. The reference solution is computed using a Strang-splitting scheme with *dt*_1D_ = 0.25 μs, yielding *V*_m_(0.1 ms) ≈ −23.5219mV.

**Figure 4:**
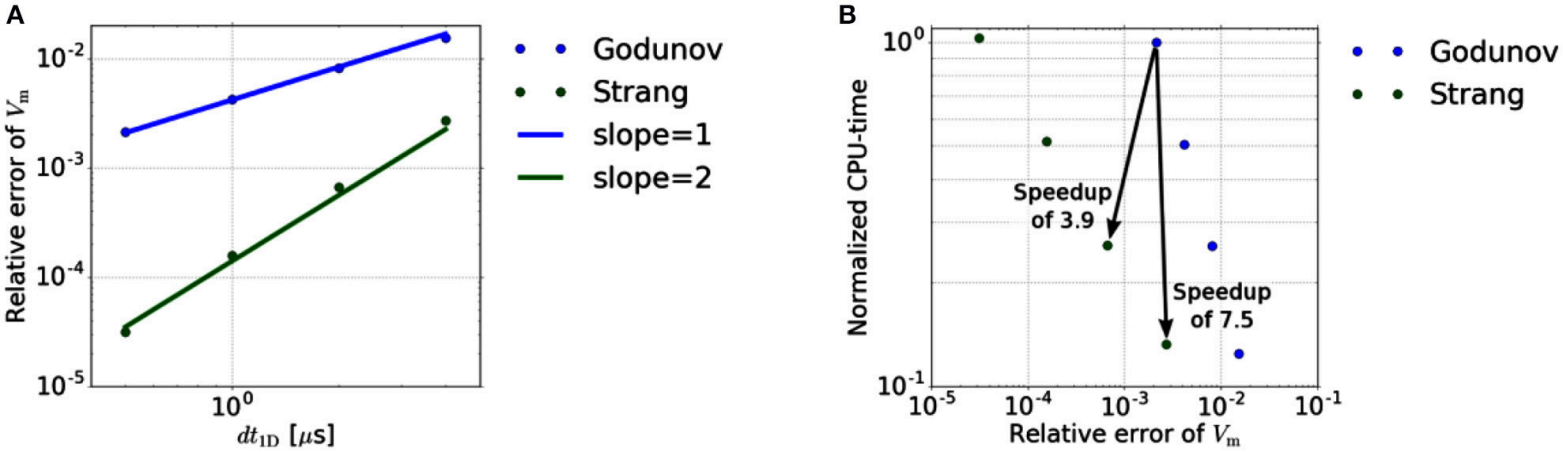
**(A)** Relative error dependency on the 1D time step size *dt*_1D_. The error of the Godunov- and Strang-splitting scheme shows the expected $O\left(dt_{1\text{D}}\right)$ and $O\left(dt_{1\text{D}}^{2}\right)$ behavior, respectively. **(B)** Efficiency of different splitting schemes. Each scheme is performed for *dt*_1D_ = 0.5, 1, 2 and 4 μs.

Figure 4A shows the relative errors of *V*_m_(0.1 ms) at a stimulated sub-cell for the Godunov- and Strang-splitting schemes. Comparable relative errors as for the Godunov scheme with *dt*_1D_ = 0.5 μs are achieved for the Strang splitting scheme with *dt*_1D_ = 2 or 4 μs. Qu and Garfinkel (1999) applied the Strang splitting scheme on the monodomain equation in cardiac conduction, using a different reaction term than in this work. However, it is not entirely clear whether second order convergence is exhibited by their numerical experiments. For an electrocardiogram simulation Sundnes et al. (2005) used the same scheme on the more general bidomain equation, achieving a nearly second order scheme. In contrast to these works our results show a true second-order error dependency. The resulting speedups are depicted in Figure 4B by arrows pointing from Godunov to Strang data points. There, the compute times are normalized with respect to the compute time of the Godunov scheme for *dt*_1D_ = 0.5 μs.

Based on a relative error in *V*_m_ of about 2·10^−3^, the improved time stepping scheme achieves a speedup of 7.54, if the accuracy requirement is weakened slightly. If the error constraint is not weakened, we still obtain a speedup of 3.89. Note that, for more restrictive error limits, the speedup achieved with a second-order scheme will be even higher due to the higher convergence order.

#### 3.2.3. Solving the linear systems of equations in the 1D model

**Figure 5:**
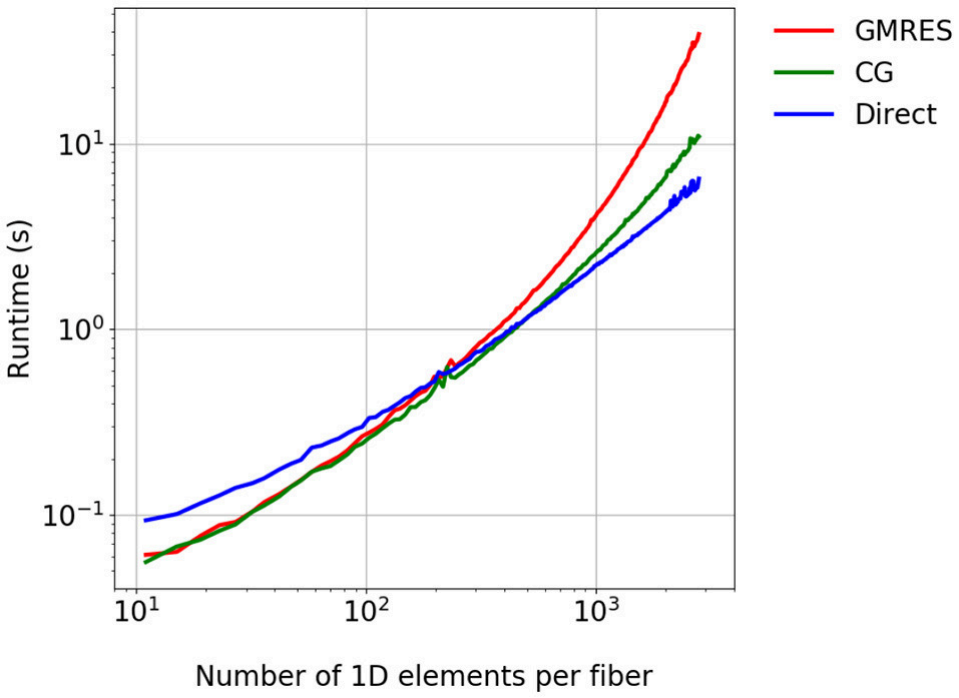
Comparison of the runtime for different linear solvers. A single fiber is considered for the time *t* ∈ [0, 3 ms].

In a further experiment, which solves a 1D diffusion problem, we consider a single fiber inside one 3D element for the time interval *t* ∈ [0, 3 ms]. The Godunov splitting scheme is employed with time step sizes $dt_{1\text{D}}=5\cdot 10^{-3}\text{ms}$ and $dt_{0\text{D}}=10^{-4}\text{ms}$, as the experiment is largely independent of the splitting scheme. We compare the GMRES solver with 30 restarts against the CG solver and a direct solver from the MUMPS library. Figure 5 shows the expected reduction in the runtime for the CG and direct solvers. Although the direct solver has a higher runtime for a small number of 1D elements, it requires the lowest runtime for finer discretizations and shows a linear complexity with the number of 1D elements.

#### 3.2.4. Runtime analysis during serial execution of the full model

In previous sections we considered subproblems of the computational model. In this section we measure the overall effect of the combined improvements. A complete single-twitch scenario as described in section 3.1 is simulated for a time span of [0, 1 ms]. We compare all numerical and algorithmic improvements of this paper against the baseline setting of Heidlauf and Röhrle (2013).

The 3D spatial discretization comprises 8 Taylor-Hood finite elements containing 36 muscle fibers (*n*_*x*_ = *n*_*y*_ = 6) in total. For the baseline setting using the Godunov splitting scheme the time steps are set to *dt*_3D_ = 1 ms, $dt_{1\text{D}}=5\cdot 10^{-4}\text{ms}$ and $dt_{0\text{D}}=10^{-4}\text{ms}$, i. e., *N* = 2000 and *K* = 5. For the Strang splitting scheme the values are *dt*_3D_ = 1 ms, $dt_{1\text{D}}=4\cdot 10^{-3}\text{ms}$ and $dt_{0\text{D}}=2\cdot 10^{-3}\text{ms}$, i. e., *N* = 250 and *K* = 2. For the baseline setting the linear system of equations arising from the 1D problem is solved using a restarted GMRES solver with a restart after 30 iterations and relative residual tolerance of 10^−5^. The improved simulation uses the direct solver as described in section 2.2.2.3. To solve the 3D problem, Newton's method from the PETSc library is used with a relative and absolute tolerance of 10^−8^ and a backtracking line search approach with a maximum number of 40 iterations.

To assess problem size scalability, we vary the number of 1D elements along each muscle fiber and measure the runtimes of the simulation components. Note that the number of sub-cellular model instances is changed accordingly.

**Figure 6:**
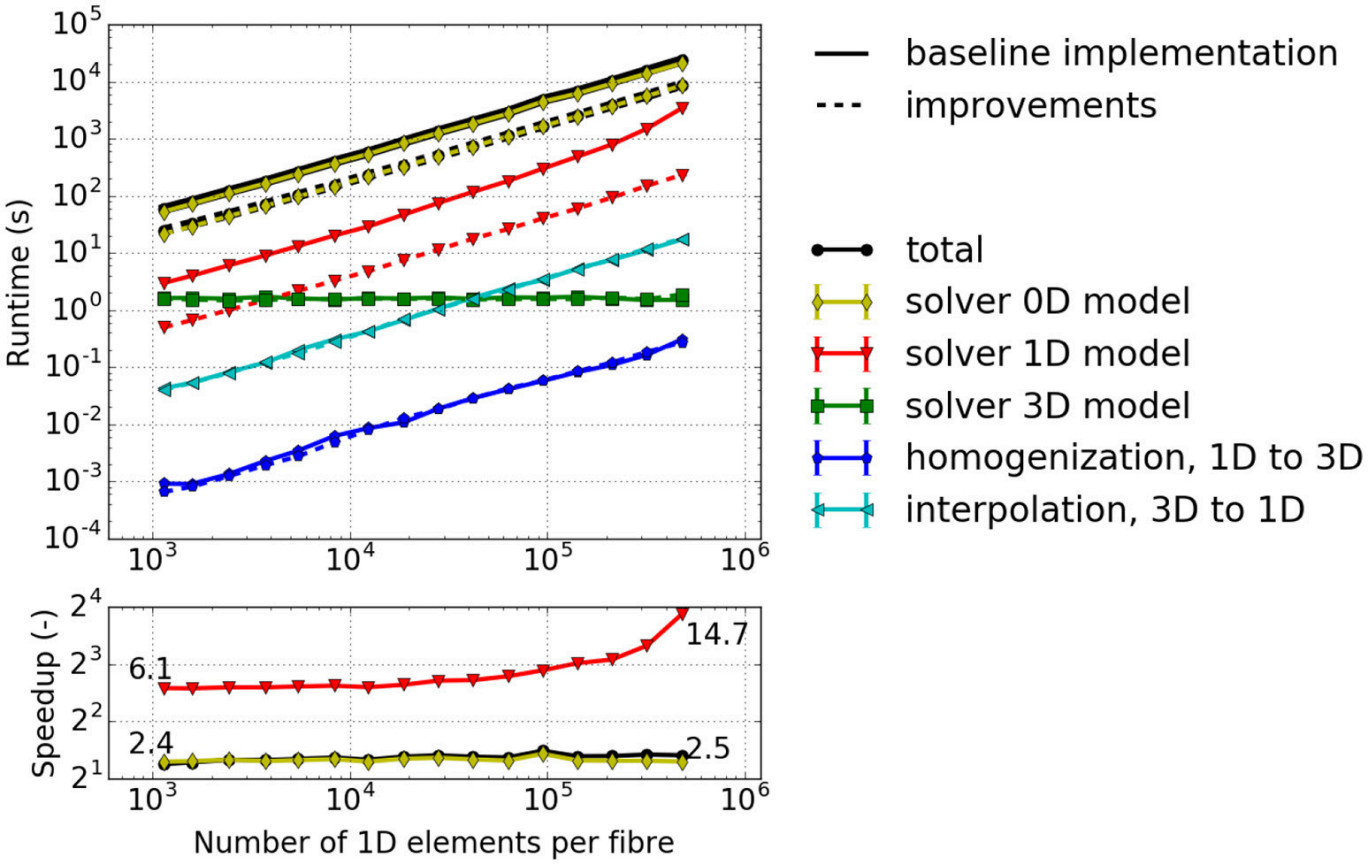
Runtime for a simulated time interval *t* ∈ [0, 1 ms] with a varying number of elements per fiber. 2 × 2 × 2 3D elements, 6 × 6 1D fibers. Solid lines: baseline implementation, Dashed lines: implementation with improvements of this paper.

The results depicted in Figure 6 provide the following insights: (i) The majority of the runtime is spent solving the 0D problem. (ii) The portion of runtime spent solving the 3D problem is negligible. This is due to the low number of 3D finite elements for the mechanics problem. Realistic models would, however, require a finer resolution of the 3D problem. (iii) The runtime for the other computational components increases approximately linearly with the number of fiber elements. This indicates a good scaling behavior with respect to problem size. (iv) The computations of the macroscopic variable *l*_hs_ from the fiber nodes, the homogenized activation parameter $\overset{-}{\gamma }$ (homogenization), as well as *l*_hs_ (interpolation) have almost no impact on the overall computational time. However, interpolation is more time consuming as it involves simultaneously traversing the fiber and the 3D meshes, whereas homogenization requires only a single averaging operation for each Gauss point of the 3D elements.

### 3.3. Parallel scaling experiments

In the following we conduct parallel scalability experiments to investigate the behavior of the simulation on highly parallel compute clusters. All experiments are conducted on HazelHen, the Tier-1 supercomputer at the High Performance Computing Center Stuttgart (HLRS). A dual-socket node of this Cray XC40 contains two Intel® Haswell E5-2680v3 processors with base frequency of 2.5 GHz, maximum turbo frequency of 3.3 GHz, 12 cores each and 2 hyperthreads per core, leading to a total number of 48 possible threads per node. We present the results of a strong scaling (Experiment #1) and weak scaling experiments (Experiments #2 and #3) as well as an investigation of partitioning strategies (Experiment #4).

#### 3.3.1. Strong scaling measurements—experiment #1

**Figure 7:**
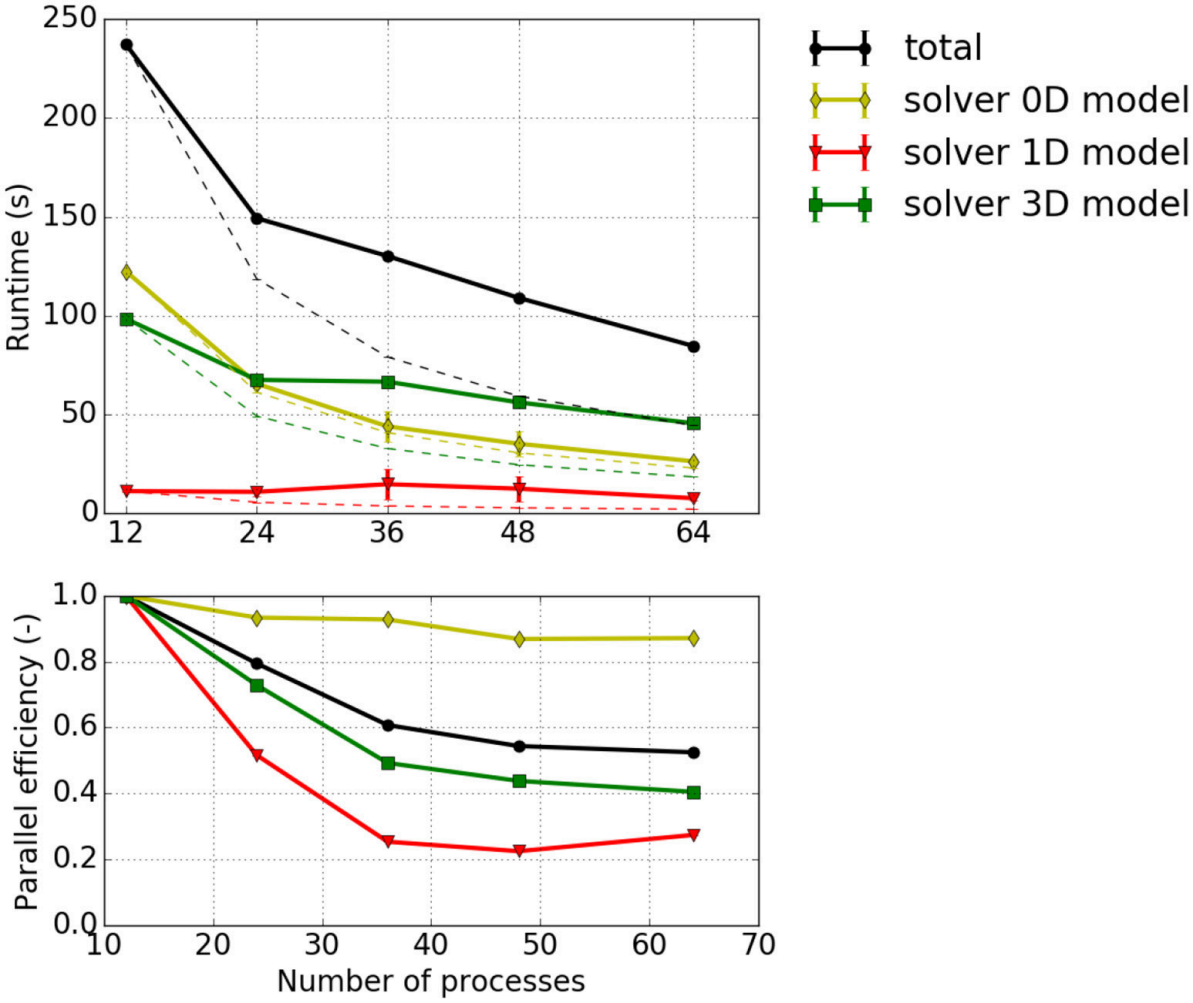
Strong scaling measurements—Experiment #1: Scenario with 12 × 12 × 8 = 1,152 3D elements, 24 × 24 fibers (i.e., 13,824 1D elements), the cube shaped partitioning strategy computed with 1–4 nodes with 12 processes per node. **(Top)** Total runtime and runtimes of solvers for the 0D, 1D and 3D problem, *T*_*p*_, (solid lines); projected runtimes for optimal scaling, *T*_*p*, opt_ = *T*_12_·*p*/12 (dashed thin lines). **(Bottom)** Parallel efficiency *E*_*p*_ = *T*_*p*, opt_/*T*_*p*_.

Strong scaling investigates the runtime for a fixed problem size with respect to different process counts. Figure 7 depicts strong scaling results for the specified problem with 13,824 1D elements. Taking the first runtime measurement with 12 processes (*T*_12_) as reference, the parallel efficiency for a process count *p* is computed from the runtime *T*_*p*_ as *E*_*p*_ = (*T*_12_/*T*_*p*_)·(*p*/12) and visualized in the bottom plot of Figure 7. It can be seen that the 0D model solver shows a good parallel efficiency of more than 80% whereas the parallel efficiencies for the 3D solver and the 1D solver drop below 50 and 30%, respectively. This matches the fact that the half-sarcomere sub-models (0D) are completely independent of each other whereas the solutions of 3D and 1D problems require communication.

#### 3.3.2. Weak scaling measurements—experiment #2

For weak scaling, the problem size is increased proportional to the number of processes. Thus, invariants are the number of elements per process and the overall shape of the computational domain. Here, we show weak scaling for both partitioning strategies: partitioning only in *y*- and *z*-direction, i.e., pillar-like partitioning, and cuboid partitioning. We start with 24 processes on a single node of HazelHen with an initial partition consisting of *p*_*x*_ × *p*_*y*_ × *p*_*z*_ = 1 × 6 × 4 = 24 subdivisions for both pillar-like and cuboid partitioning. Each partition contains *e*_*x*_ × *e*_*y*_ × *e*_*z*_ = 2 × 2 × 2 = 8 3D elements per MPI rank. Further, we ensure that each 3D element contains 2 × 2 fibers in *x*-direction with three 1D elements per fiber, i.e., 12 1D elements per 3D element. Hence, the initial problem is made up of 24 × 8 = 192 elements and 12 × 8 × 4 = 384 fibers.

In the series of measurements for the two partitioning strategies, further subdivisions are defined such that the pillar-like or cuboid partitioning structure is maintained. The refinements are obtained by first refining by a factor of 2 in the *x*-direction, in the *z*-direction and then in the *y*-direction before repeating the process. For the cuboid partitioning, we fix the number of 3D elements that each MPI rank contains to be 2 × 2 × 2. For the pillar-like partitioning, the constraint is that each sub-domain spans over all three-dimensional elements in the *x*-direction, whose number varies with increasing problem size. Therefore, the number of elements per MPI rank in *y*- and *z*-direction is halved for each refinement in an alternating way. This way, we double the number of partitions while maintaining the constant number of eight 3D elements per MPI rank. By allocating 24 processes on the 24 cores of each node (no hyperthreading), we scale from 1 to 32 nodes, i. e., from 24 to 768 cores. Table 1 provides the details on the partitioning and the number of three-dimensional and one-dimensional elements.

**Table 1:** Weak scaling measurements–experiment #2: Problem and partition sizes for 1 to 32 nodes with 24 processes per node, i.e., 24–768 cores of HazelHen.

**Nodes**	**3D Elements**	**1D El**.	**Pillars**		**Cubes**	

	*p*_*x*_*e*_*x*_×*p*_*y*_*e*_*y*_×*p*_*z*_*e*_*z*_		*p*_*x*_×*p*_*y*_×*p*_*z*_	*e*_*x*_×*e*_*y*_×*e*_*z*_	*p*_*x*_×*p*_*y*_×*p*_*z*_	*e*_*x*_×*e*_*y*_×*e*_*z*_
1	2 × 12 × 8	2,304	1 × 6 × 4	2 × 2 × 2	1 × 6 × 4	2 × 2 × 2
2	4 × 12 × 8	4,608	1 × 6 × 8	4 × 2 × 1	2 × 6 × 4	2 × 2 × 2
4	4 × 12 × 16	9,216	1 × 12 × 8	4 × 1 × 2	2 × 6 × 8	2 × 2 × 2
8	4 × 24 × 16	18,432	1 × 12 × 16	4 × 2 × 1	2 × 12 × 8	2 × 2 × 2
16	8 × 24 × 16	36,864	1 × 24 × 16	8 × 1 × 1	4 × 12 × 8	2 × 2 × 2
32	8 × 24 × 32	73,728	1 × 24 × 32	8 × 1 × 1	4 × 12 × 16	2 × 2 × 2

**Figure 8:**
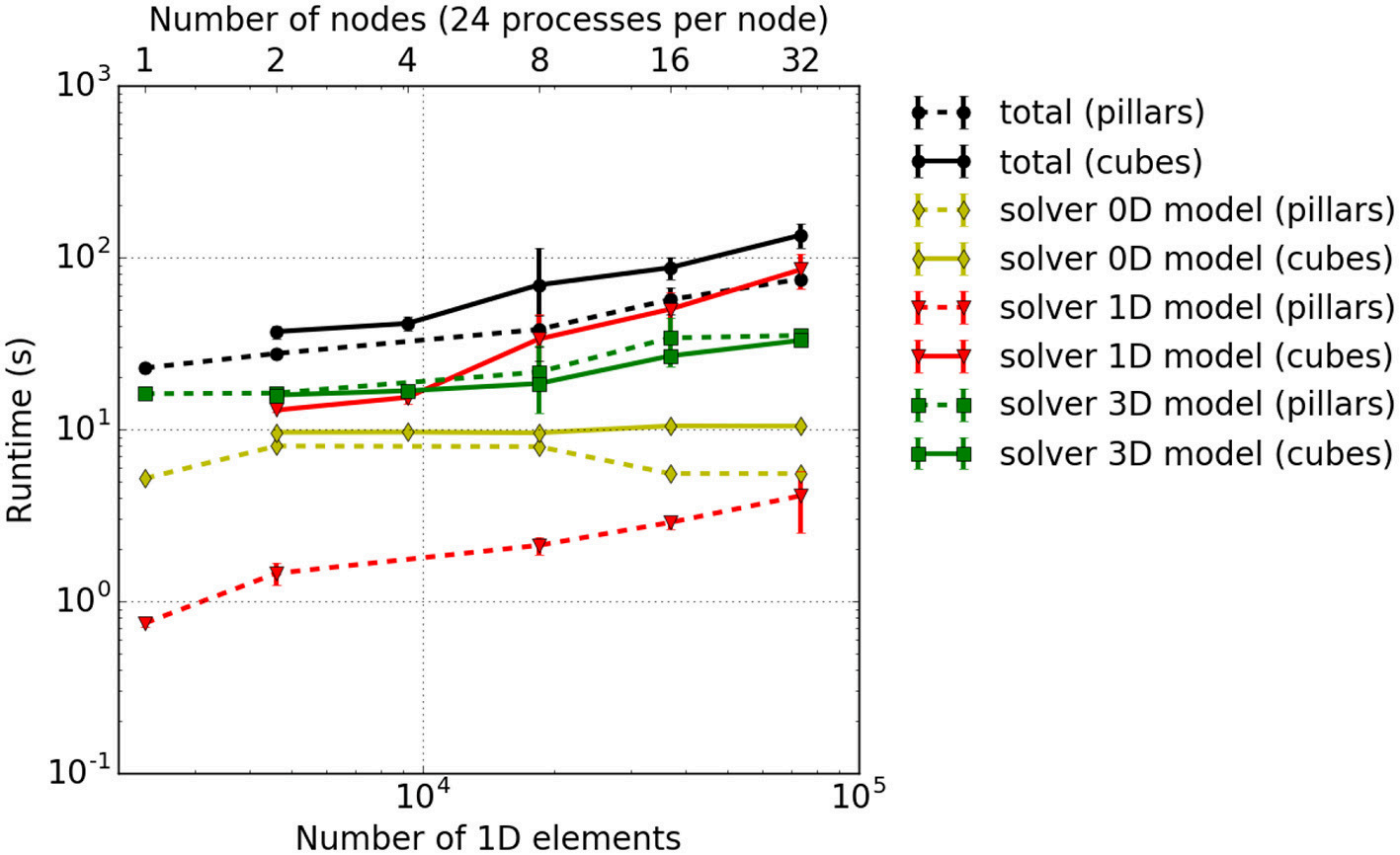
Weak scaling measurements–experiment #2: Total runtime as well as individual runtimes (solver for the 0D, 1D, and 3D problem) for the cuboid partitioning (solid lines) and pillar-like partitions (dashed lines). The error bars indicate estimated standard deviation of runtimes on all involved processes of a single simulation run.

Results are shown in Figure 8, and show that the solver for the 3D model has a slightly higher computational time for the pillar-like partitioning compared to the cuboid partitioning. This is expected as the partition boundaries are larger and induce more communication. For the 1D problem solver, pillars are better as fibers are not subdivided between multiple cores and no communication is needed. The reduced benefit from a cuboid partitioning is due to the fact that the time spent on communication is rather dominant compared to the time needed to solve the rather small problem, e. g., only 3*e*_*x*_ = 6 1D elements of a fiber are locally stored in each partition. This should improve as one chooses larger sub-problem sizes, i. e., increases the number of nodes per fiber.

Theoretically, the time needed to solve the 0D problem should not be affected by the domain decomposition. However, due to cache effects, the runtime for a cuboid partitioning is slightly higher. Overall, this leads to a higher total computational time for cuboid partitioning compared to the pillar-like partitioning. This conclusion is, however, only valid for the chosen scenario and for the relatively low number of cores. Note that extending this scaling experiments to a larger numbers of cores is currently limited due to memory duplications in the current code. This needs to be first eliminated before conducting further scaling studies.

#### 3.3.3. Weak scaling measurements – experiment #3

**Figure 9:**
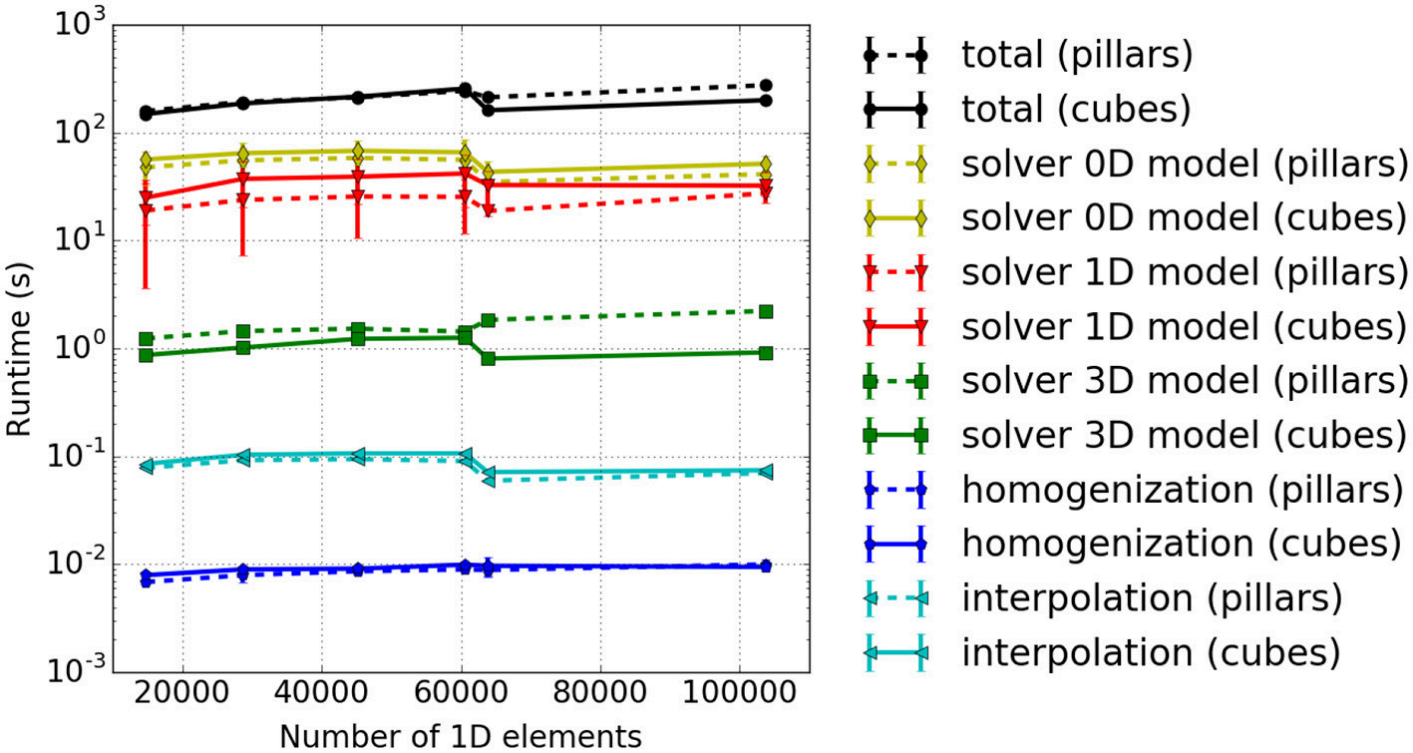
Weak scaling measurements—experiment #3: Runtimes for different model components. The results for cuboid and pillar-like partitions are depicted by solid and dashed lines, respectively. Different runtime components are encoded in colors, i. e., the total runtime in black, 0D solver in yellow, the 1D solver in red, the 3D solver in green, the interpolation in light blue and the homogenization in dark blue.

While the somewhat artificial setting in experiment #2 yields perfect pillar-like or cuboid partitions, experiment #3 addresses a more realistic setup, where we increase the number of processes more smoothly, i.e., by less than a factor of two in each step. With this, it is not possible anymore to choose perfect cuboid or pillar-like partitions. Thus, we identify reasonable parameters by solving an optimization problem that trades the targeted aspect ratio of sub-domain shape against process counts. Note that the combination of the number of processes and the number of elements leads to partitions at the boundary of the computational domain that potentially have less elements than interior partitions. Compared to the previous example, the number of 3D elements per process is here only approximately constant, with the pillar-like partitions getting closer to constant size than the cuboid ones. The numbers of processes and the dimensions of the computational domain are listed in Table 2. Figure 9 presents the runtime results.

**Table 2:** Weak scaling measurements—experiment #3: Number of elements, number of fibers and partition sizes.

**Nodes**,	**3D Elements**	**1D El**.	**Pillars**		**Cubes**	

**Cores**	*p*_*x*_*e*_*x*_×*p*_*y*_*e*_*y*_×*p*_*z*_*e*_*z*_		*p*_*x*_×*p*_*y*_×*p*_*z*_	*e*_*x*_×*e*_*y*_×*e*_*z*_	*p*_*x*_×*p*_*y*_×*p*_*z*_	*e*_*x*_×*e*_*y*_×*e*_*z*_
1, 24	16 × 11 × 7	14,784	1 × 6 × 4	16 × 2 × 2	4 × 3 × 2	4 × 4 × 4
2, 40	18 × 19 × 7	28,728	1 × 10 × 4	18 × 2 × 2	4 × 5 × 2	5 × 4 × 4
3, 60	18 × 19 × 11	45,144	1 × 10 × 6	18 × 2 × 2	4 × 5 × 3	5 × 4 × 4
4, 84	17 × 27 × 11	60,588	1 × 14 × 6	17 × 2 × 2	4 × 7 × 3	5 × 4 × 4
6, 140	38 × 20 × 7	63,840	1 × 20 × 7	38 × 1 × 1	10 × 7 × 2	4 × 3 × 4
8, 192	45 × 16 × 12	103,680	1 × 16 × 12	45 × 1 × 1	12 × 4 × 4	4 × 4 × 3

As already discussed above, the ODE solver for the 0D-problem (yellow line) requires the majority of the runtime. This is followed by the solution times for the 1D (red line) and 3D (green line) sub-problems. The blue lines depict the duration of the interpolation and homogenization between the node positions of the 1D fibers and the 3D mesh. It can be seen that the computational times stay nearly constant for increasing problem size. As in the previous experiment, the 3D solver performs better for cuboid partitioning whereas the 1D solver is faster for pillar-like partitions. In this scenario, the cuboid partitioning slightly outperforms the pillar-like partitioning, as expected.

**Figure 10:**
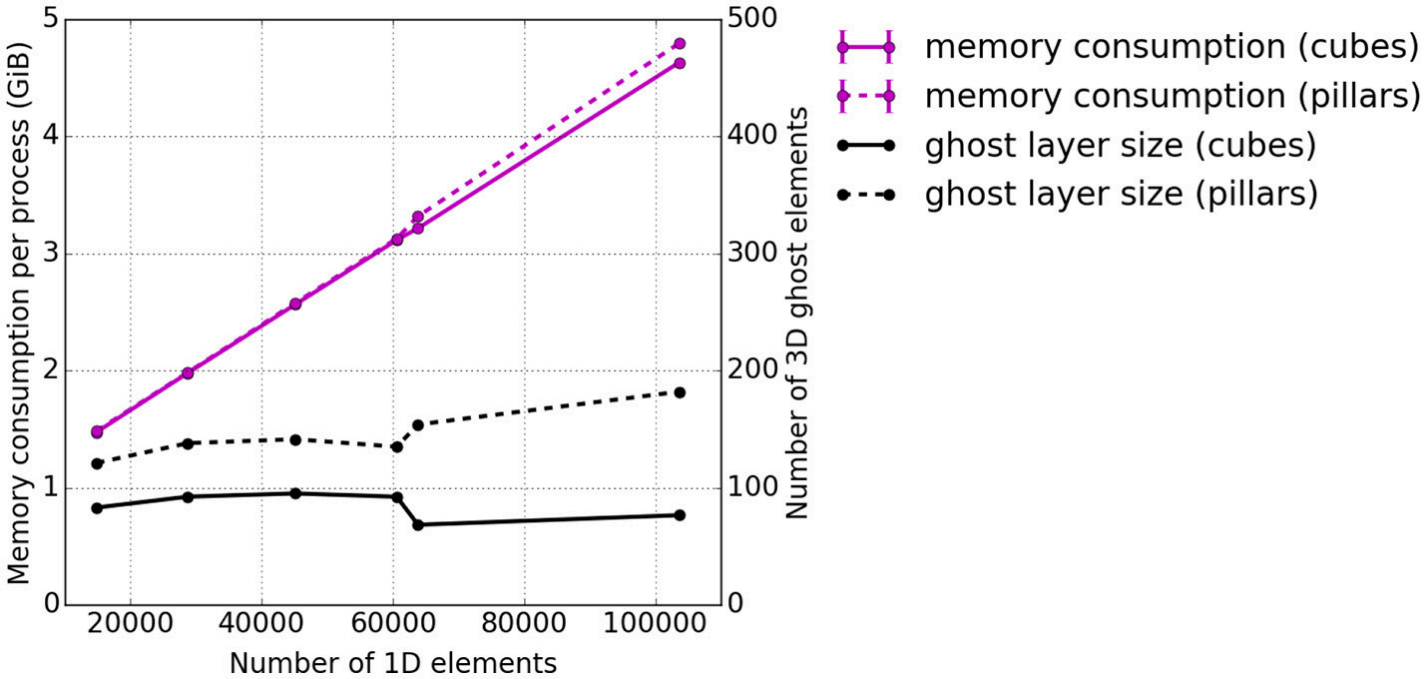
Weak scaling measurements—experiment #3: Total memory consumption per process at the end of the runtime. The total memory consumption is depicted in magenta and the average number of 3D ghost layer elements per process in black. Again, the solid lines represent cuboid partitioning and the dashed line piller-like partitioning.

As before, the memory consumption appears to be a weakness. Therefore, additional tests investigating the memory consumption per process at the end of the runtime were carried out. The memory consumption for the presented scenario is plotted in Figure 10 with respect to the overall number of 1D elements. Also the average number of ghost layer elements per process is depicted. Ghost layer elements are copies of elements adjacent to the partition of a process, i.e., they belong to the subdomain of a neighboring process. They are used as data buffers for communication. We observe that the average number of ghost elements per process for the 3D problem is higher for pillar-like partitions (dashed black line) than for the cuboid partitions (solid black line). A sharp increase of memory consumption (magenta lines) is observed independent of the partitioning scheme. This is due to duplications of global data on each process, which will be eliminated in future work. Compared to this effect, the difference between the number of ghost elements needed for the two partitioning strategies is negligible.

#### 3.3.4. Dependency between runtime and partition shape – experiment #4

**Figure 11:**
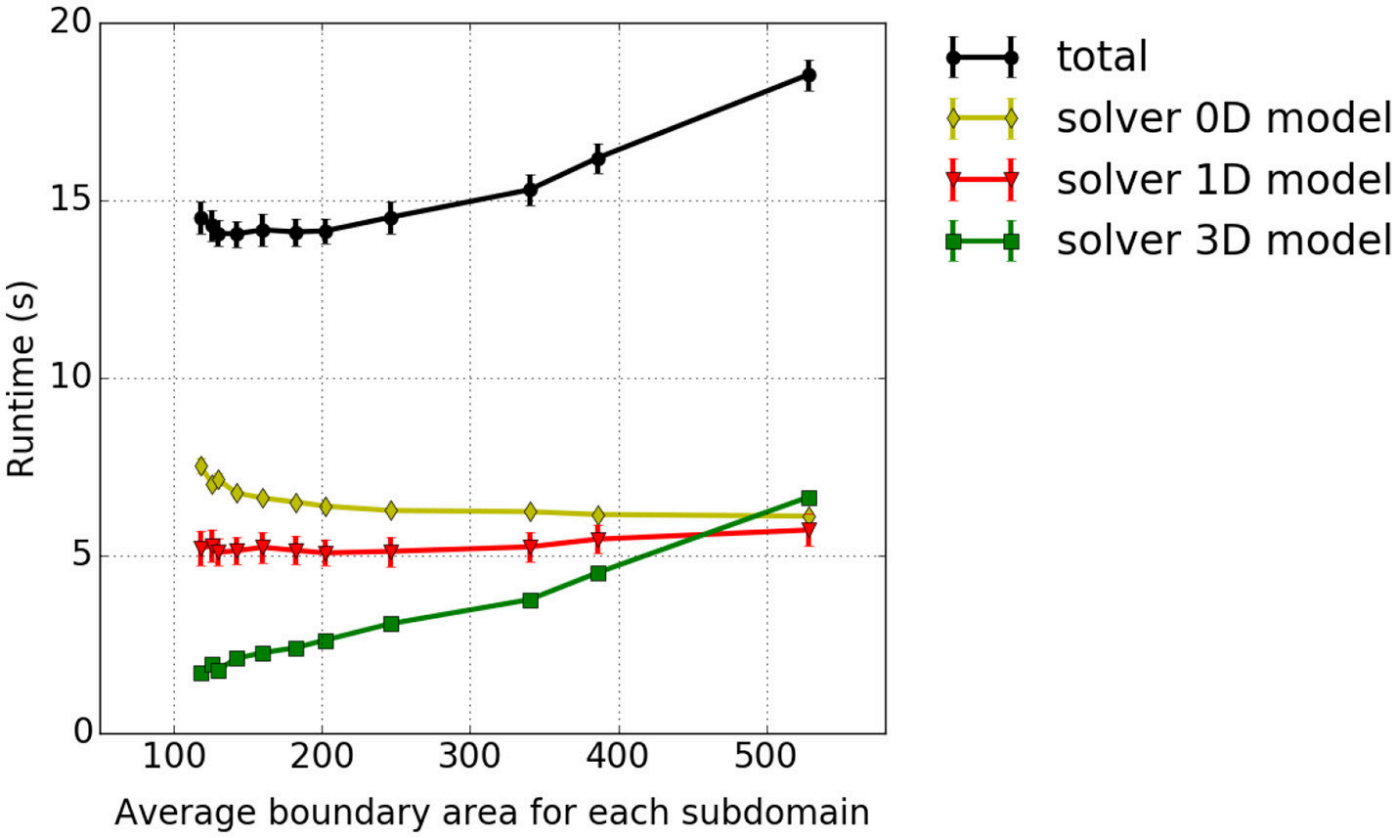
Dependency between runtime and partition shape—experiment #4: Runtime in dependence on the average boundary area of the partitions. We show the accumulated total computational time and the runtimes of the sub-problems as in the previous studies.

In our fourth scaling test, the dependency of the solver of the 3D continuum-mechanical problem on the partitioning strategy is investigated. We analyse how different domain decomposition approaches, in particular approaches other than the previously discussed pillar-like and cuboid partitioning schemes, affect the runtime. A test case with 144 × 12 × 12 three-dimensional elements is considered. The setup, otherwise, is as described in section 3.1. To reduce the contributions of the 0D/1D sub-problem and focus on the performance of the 3D components, we include in each 3D element only two 1D fiber elements. The domain is decomposed into a constant number of 144 partitions by axis-aligned cutplanes in all possible ways. To distinguish between the different partitioning variants, we compute the average boundary surface area between the partitions for each variant and relate this to runtime. The results are presented in Figure 11. The smallest average surface area between the partitions, which corresponds to the first data point in Figure 11, is obtained for a partitioning with 144 partitions with 4 × 6 × 6 elements each. The highest average surface area between the partitions, which is the last data point within Figure 11, is obtained for 144 partitions with 1 × 12 × 12 elements each. All experiments are run on 12 nodes of Hazel Hen with 12 processes per node. It can be seen that only the time needed to solve the 3D continuum-mechanical problem increases monotonically with respect to the average surface area between the partitions, i. e., depends on the partitions' shape. This is expected. Further, the runtime ratio of the 3D solver between the partitioning with the smallest and largest average surface area is 1:4.3.

### 3.4. Visualization results

In this section, we describe the results obtained using our new ray-tracing-based visualization within the MegaMol system. Our goal is to demonstrate the capabilities of our rendering approach for the interactive visualization of complex, real-world simulation data sets. Therefore, we used data from previous simulations to showcase these visualization capabilities, in particular, data from the Tibialis Anterior simulation performed by Heidlauf and Röhrle (2013). Analyzing and optimizing existing code for HPC infrastructures is best performed with test cases for which the geometry has a minimal influence. Under this consideration, the cuboid muscle test case introduced in section 2.1.4 would have been an obvious choice. However, in contrast to the Tibialis Anterior data, the cuboid muscle test case is too small and simple to demonstrate the full capabilities of our new visualization approach for complex geometries.

**Figure 12:**
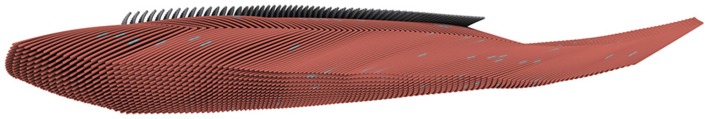
The discretized 1D muscle fibers are rendered as continuous tubes to show the characteristics and implicit geometry (with the distinct fiber directions of the superficial and deep part of the Tibialis Anterior) of the individual strands. The color coding shows the distribution of parameter values along the fibers (local membrane potential; red: low, blue: high).

Our test data set consists of 3,600 fibers, which are discretized into a total of 144,000 1D elements. The consecutive elements along each fiber are connected via tubes to visualize the fibers. Figure 12 shows a rendering created by MegaMol (Grottel et al., 2015) using our integration of the CPU ray tracing engine OSPRay (Wald et al., 2017). Color is used to illustrate values of the elements, in this case the local membrane potential. The interactive ray tracing offers very high image quality, including global illumination effects that increase the perception of spatial details. This is especially visible with the shadows between fibers, which help to perceive the distance between them as well as deformations of the individual fibers with respect to their neighbors. That is, our visualization approach not only delivers publication-quality images, which is often not possible for interactive visualization of large data using classical rendering approaches, but it is also beneficial for the visual analysis of local details as well as the overall spatial impression of the data.

**Figure 13:**
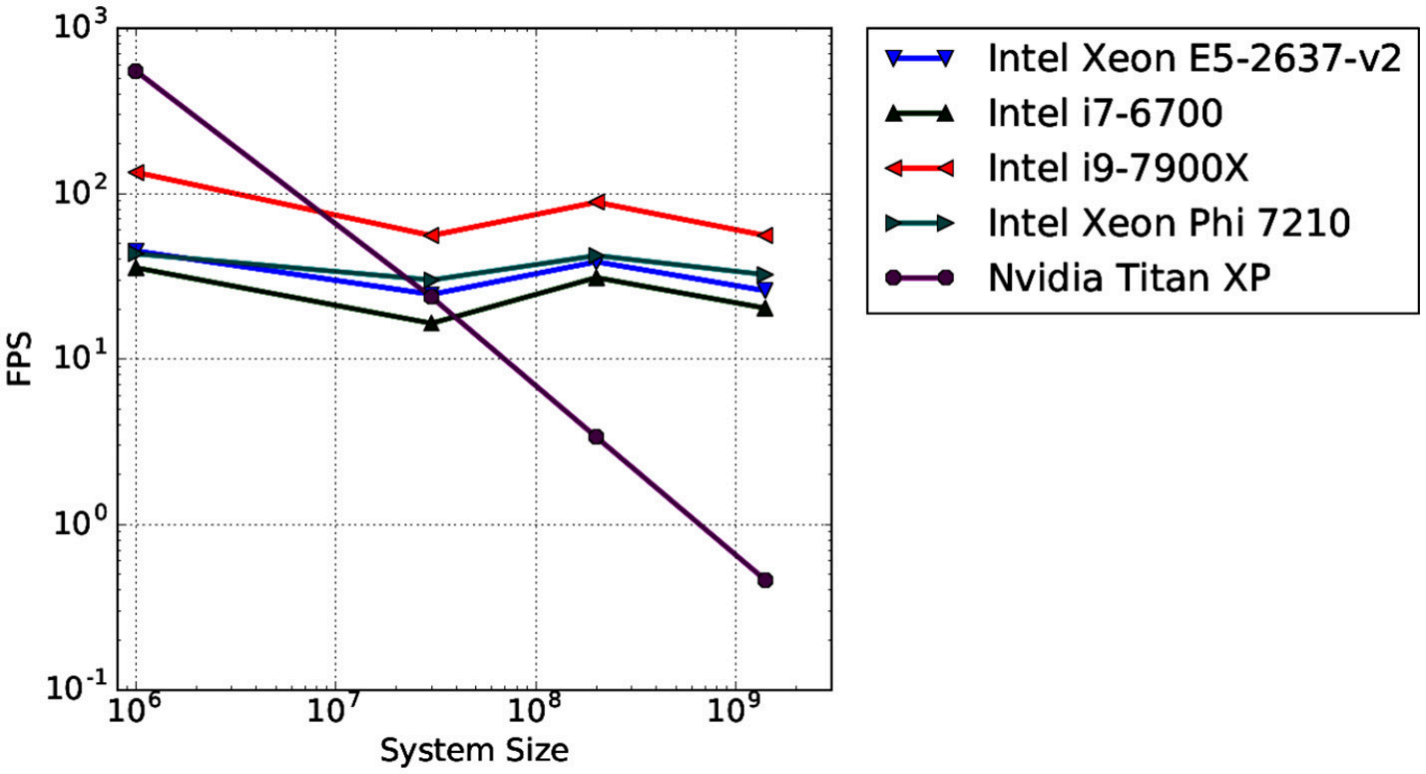
Average rendering performance (frames per second, FPS) for four different data sets measured on four different CPU architectures (blue, green, red, cyan; triangle markers). For reference, the rendering performance of a GPU-based ray casting measured on a high-end GPU is provided (violet; circle marker).

To test the scaling behavior of our OSPRay integration into MegaMol, we measured the rendering performance of four different-sized systems. We used synthetic data sets ranging from 10^6^ to 1.4 · 10^9^ elements rendered as sphere geometries. Spheres are the most basic visualization primitive and can be rendered very fast, therefore, they are typically used as a baseline case for performance tests using large data sets. We also compare the CPU ray tracing performance with a GPU-based ray casting, which is a fast and efficient way to render large numbers of particles (e.g., Reina and Ertl, 2005). The CPU ray tracing uses a P-k-d tree by Wald et al. (2015) for fast ray traversal. This tree is a memory-efficient hierarchical data structure used for space partitioning. All measurements were executed on a single desktop PC at a resolution of 1280 × 720 pixels. Figure 13 shows the results obtained by different Intel CPUs for the OSPRay rendering compared to the GPU rendering on a high-end Nvidia consumer graphics card (Nvidia Titan XP). As observable, the GPU-based rendering outperforms the CPU-based ray tracing only for the smallest test case. For more than 10^7^ spheres, the OSPRay ray tracing clearly outperforms the GPU rendering. This result agrees with our earlier findings presented in (Rau et al., 2017).

In summary, the CPU-based ray tracing approach that we chose is superior to classical GPU-based rendering not only in terms of image quality but also in terms of scalability for very large data sets. This is important for the visual analysis of HPC simulation data, which constantly increases in size as well as complexity due to improvements in simulation codes as well as the availability of faster HPC hardware. Our results demonstrate that real-time ray tracing is a viable solution nowadays for rendering large muscle fiber simulation data sets compared to classical rasterization-based approaches. It delivers not only superior image quality, which is beneficial for visual analysis, but also higher rendering performance even on single desktop PCs.

## 4. Discussion

Using models to gain new insights into the complex physiological or anatomical mechanisms of biological tissue, or to better interpret and understand experimentally measured data, requires accurate and detailed models of the underlying mechanisms. This can lead very quickly to highly complex and computationally extremely demanding models. Software packages such as OpenCMISS are designed to build up computational models for a variety of complex biomechanical systems, e.g., for the chemo-electromechanical behavior of skeletal muscles after recruitment, the mechanics of the heart, the functioning of the lung, etc. Such software packages might already run within a parallel computing environment, but are not necessarily optimized to run large-scale simulations on large-scale systems such as HazelHen, the Tier-1 system in Stuttgart. Thus, before being able to exploit the full capabilities of supercomputers, they have to be analyzed and optimized to achieve good scaling properties—ideally perfect scaling meaning that the simulation of a twice as large problem on twice as many nodes/cores requires the same runtime as the original setup.

Within this paper, we have demonstrated that the chemo-electromechanical multi-scale skeletal muscle model as introduced in section 2.1 and implemented in OpenCMISS is capable of running significant large-scale model setups in a parallel compute environment. We have simulated the deformation of a skeletal muscle in which 34, 560 randomly activated fibers are discretized with 103, 680 1D elements. Due to the algorithmic optimizations a meaningful compute time reduction was achieved. Further, by utilizing a standard test case, we have been able to show good strong and weak scaling properties for a small number of compute nodes. For the partitioning of the domain, two different approaches have been considered: a pillar-like partition along fiber directions and a minimal-surface partitioning. The solution times of the 3D and the 1D solver mainly depend on the domain partitioning. The 1D solver profits from pillar-like partitions, while the 3D solver exhibits lower runtimes for cube-like minimal-surface partitions. In addition to its advantage in terms of communication complexity for large numbers of parallel processes, the minimal-surface domain decomposition strategy investigated here is generalizable to arbitrary geometry settings even for unstructured meshes based, for example, on graph-partitioning methods.

However, for more realistic large-scale simulations, further aspects concerning the model, algorithms, implementation, and visualization need to be considered: a more complicated chemo-electromechanical model that includes, for example, the mechanical behavior of titin (Heidlauf et al., 2016, 2017) and further important biophysical details such as metabolism, a biophysical recruitment model (Heidlauf et al., 2013), and a feedback mechanism from the spindles and the golgi-tendon organs to the neuromuscular system; simulation and visualizing of the surface EMG to further test motor unit decomposition algorithms; novel or custom-tailored efficient numerical schemes for new model components and coupling with the existing ones; or integrating chemo-electro-mechanical modeling approaches to extend forward simulations using continuum-mechanics musculoskeletal system models Röhrle et al. (2017) in order to drive them not only through optimization Valentin et al. (2018) but also by means of neural recruitment, and hence obtain a deeper insight into neuromuscular recruitment principles.

Our goal is to set up large-scale simulations for a single chemo-electromechanical skeletal muscle model with a realistic number of fibers (e. g., about 300,000) of realistic length. The results of these simulations need to be visualized and analyzed for which we extend MegaMol to offer novel, comprehensive visualizations that allow users to interactively explore the complex behavior of muscle fiber simulation data. We will validate our simulation by comparisons of the simulated surface EMG of a muscle with experimental data obtained via non-invasive and clinically available diagnostic tools. Finally, our simulations can serve as a new tool to investigate the interplay of the underlying complex and coupled mechanisms leading from neural stimulation to force generation.

## Author contributions

All authors have equally contributed to the conception and design of the work, data analysis and interpretation, drafting of the article, and critical revision of the article. Hence, the author list appears in alphabetical order. In addition NE, TK, AK, BM, and TR have conducted the simulations and summarized their results. All authors fully approve the content of this work.

### Conflict of interest statement

The authors declare that the research was conducted in the absence of any commercial or financial relationships that could be construed as a potential conflict of interest.

## References

[B1] Ahrens J. Geveci B. Law C. (2005). ParaView: an end-user tool for large data visualization, in Visualization Handbook, eds Hansen C. Johnson C. R. (Burlington: Butterworth-Heinemann), 717–731.

[B2] Amestoy P. R. Duff I. S. L'Excellent J.-Y. Koster J. (2001). A fully asynchronous multifrontal solver using distributed dynamic scheduling. SIAM J. Matrix Anal. Appl. 23, 15–41. 10.1137/S0895479899358194

[B3] Amestoy P. R. Guermouche A. L'Excellent J.-Y. Pralet S. (2006). Hybrid scheduling for the parallel solution of linear systems. Parallel Comput. 32, 136–156. 10.1016/j.parco.2005.07.004

[B4] Balay S. Abhyankar S. Adams M. F. Brown J. Brune P. Buschelman K. (2015). PETSc Users Manual. Technical Report ANL-95/11 - Revision 3.6, Argonne National Laboratory.

[B5] Balay S. Gropp W. D. McInnes L. C. Smith B. F. (1997). Efficient management of parallelism in object oriented numerical software libraries, in Modern Software Tools in Scientific Computing, eds Arge E. Bruaset A. M. Langtangen H. P. (Basel: Birkhäuser Press), 163–202.

[B6] Blemker S. S. Pinsky P. M. Delp S. L. (2005). A 3D model of muscle reveals the causes of nonuniform strains in the biceps brachii. J. Biomech. 38, 657–665. 10.1016/j.jbiomech.2004.04.009 15713285

[B7] Böl M. Reese S. (2008). Micromechanical modelling of skeletal muscles based on the finite element method. Comput. Methods Biomech. Biomed. Eng. 11, 489–504. 10.1080/10255840701771750 19230146

[B8] Bradley C. P. Bowery A. Britten R. Budelmann V. Camara O. Christie R.. (2011). OpenCMISS: a multi-physics & multi-scale computational infrastructure for the VPH/Physiome project. Progr. Biophys. Mol. Biol. 107, 32–47. 10.1016/j.pbiomolbio.2011.06.015 21762717

[B9] Childs H. Brugger E. Whitlock B. Meredith J. Ahern S. Pugmire D. (2012a). VisIt: an end-user tool for visualizing and analyzing very large data, in High Performance Visualization: Enabling Extreme-Scale Scientific Insight, eds Wes Bethel E. Childs H. Hansen C. (New York, NY: Chapman and Hall/CRC), 357–372.

[B10] Childs H. Ma K.-L. Yu H. Whitlock B. Meredith J. Favre J. (2012b). In Situ Processing. Technical report, Ernest Orlando Lawrence Berkeley National Laboratory, Berkeley, CA.

[B11] Christie G. R. Nielsen P. M. Blackett S. A. Bradley C. P. Hunter P. J. (2009). Fieldml: concepts and implementation. Philos. Trans. R. Soc. Lond. A Math. Phys. Eng. Sci. 367, 1869–1884. 10.1098/rsta.2009.0025 19380316 PMC2665020

[B12] Cisi R. R. Kohn A. F. (2008). Simulation system of spinal cord motor nuclei and associated nerves and muscles, in a web-based architecture. J. Comput. Neurosci. 25, 520–542. 10.1007/s10827-008-0092-8 18506610

[B13] Colli Franzone P. Pavarino L. Scacchi S. (2015). Parallel multilevel solvers for the cardiac electro-mechanical coupling. Appl. Numerical Math 95, 140–153. 10.1016/j.apnum.2014.11.002

[B14] Dimitrov G. V. Dimitrova N. A. (1998). Precise and fast calculation of the motor unit potentials detected by a point and rectangular plate electrode. Med. Eng. Phys. 20, 374–381. 10.1016/S1350-4533(09)00014-9 9773690

[B15] Farina D. Merletti R. (2001). A novel approach for precise simulation of the emg signal detected by surface electrodes. IEEE Trans. Biomed. Eng. 48, 637–646. 10.1109/10.923782 11396594

[B16] Farina D. Merletti R. Stegeman D. F. (2005). Biophysics of the Generation of EMG Signals. Hoboken, NJ: Wiley-Blackwell.

[B17] Feinstein B. Lindegård B. Nyman E. Wohlfart G. (1955). Morphologic studies of motor units in normal human muscles. Cells Tissues Organs 23, 127–142. 10.1159/000140989 14349537

[B18] Fuglevand A. J. Winter D. A. Patla A. E. (1993). Models of recruitment and rate coding organization in motor-unit pools. J. Neurophysiol. 70, 2470–2488. 10.1152/jn.1993.70.6.2470 8120594

[B19] Gordon A. M. Huxley A. F. Julian F. J. (1966). The variation in isometric tension with sarcomere length in vertebrate muscle fibres. J. Physiol. 184, 170–192. 10.1113/jphysiol.1966.sp007909 5921536 PMC1357553

[B20] Grottel S. Krone M. Müller C. Reina G. Ertl T. (2015). MegaMol – A Prototyping Framework for Particle-Based Visualization. IEEE Trans. Visual. Comput. Graph. 21, 201–214. 10.1109/TVCG.2014.2350479 26357030

[B21] Gurev V. Pathmanathan P. Fattebert J.-L. Wen H.-F. Magerlein J. Gray R. A.. (2015). A high-resolution computational model of the deforming human heart. Biomech. Model. Mechanobiol. 14, 829–849. 10.1007/s10237-014-0639-8 25567753

[B22] Hawkins D. Bey M. (1994). A comprehensive approach for studying muscle-tendon mechanics. J. Biomech. Eng. 116, 51–55. 10.1115/1.2895704 8189714

[B23] Heckman C. Binder M. D. (1991). Computer simulation of the steady-state input-output function of the cat medial gastrocnemius motoneuron pool. J. Neurophysiol. 65, 952–967. 10.1152/jn.1991.65.4.952 2051212

[B24] Heidlauf T. Klotz T. Rode C. Altan E. Bleiler C. Siebert T.. (2016). A multi-scale continuum model of skeletal muscle mechanics predicting force enhancement based on actin–titin interaction. Biomech. Model. Mechanobiol. 15, 1423–1437. 10.1007/s10237-016-0772-7 26935301

[B25] Heidlauf T. Klotz T. Rode C. Siebert T. Röhrle O. (2017). A continuum-mechanical skeletal muscle model including actin-titin interaction predicts stable contractions on the descending limb of the force-length relation. PLoS Comput. Biol. 13:e1005773. 10.1371/journal.pcbi.1005773 28968385 PMC5638554

[B26] Heidlauf T. Negro F. Farina D. Rohrle O. (2013). An integrated model of the neuromuscular system, in 2013 6th International IEEE/EMBS Conference on Neural Engineering (NER) (San Diego, CA: IEEE) 227–230.

[B27] Heidlauf T. Röhrle O. (2013). Modeling the chemoelectromechanical behavior of skeletal muscle using the parallel open-source software library openCMISS. Comput. Math. Methods Med. 2013, 1–14. 10.1155/2013/517287 24348739 PMC3855958

[B28] Heidlauf T. Röhrle O. (2014). A multiscale chemo-electro-mechanical skeletal muscle model to analyze muscle contraction and force generation for different muscle fiber arrangements. Front. Physiol. 5:498. 10.3389/fphys.2014.00498 25566094 PMC4274884

[B29] Hernández-Gascón B. Grasa J. Calvo B. Rodríguez J. F. (2013). A 3D electro-mechanical continuum model for simulating skeletal muscle contraction. J. Theor. Biol. 335, 108–118. 10.1016/j.jtbi.2013.06.029 23820034

[B30] Hestenes M. Stiefel E. (1952). Methods of conjugate gradients for solving linear systems. J. Res. Natl. Bureau Stand. 49:409 10.6028/jres.049.044

[B31] Hodgkin A. L. Huxley A. F. (1952). A quantitative description of membrane current and its application to conduction and excitation in nerve. J. Physiol. 117, 500–544. 10.1113/jphysiol.1952.sp004764 12991237 PMC1392413

[B32] Johansson T. Meier P. Blickhan R. (2000). A finite-element model for the mechanical analysis of skeletal muscles. J. Theor. Biol. 206, 131–149. 10.1006/jtbi.2000.2109 10968943

[B33] Kandel E. R. Schwartz J. H. Jessell T. M. (2000). Principles of Neural Science, Vol. 4. New York, NY: McGraw-Hill.

[B34] Lafortune P. Arís R. Vázquez M. Houzeaux G. (2012). Coupled electromechanical model of the heart: parallel finite element formulation. Int. J. Numerical Methods Biomed. Eng. 28, 72–86. 10.1002/cnm.1494 25830206

[B35] Lloyd C. M. Halstead M. D. Nielsen P. F. (2004). Cellml: its future, present and past. Progress Biophys. Mol. Biol. 85, 433–450. 10.1016/j.pbiomolbio.2004.01.004 15142756

[B36] Lowery M. M. Stoykov N. S. Taflove A. Kuiken T. A. (2002). A multiple-layer finite-element model of the surface emg signal. IEEE Trans. Biomed. Eng. 49, 446–454. 10.1109/10.995683 12002176

[B37] MacIntosh B. R Gardiner P. F McComas A. J (2006). Skeletal Muscle: Form and Function, 2nd Edn. Human Kinetics.

[B38] McCallum J. B. (1898). On the Histogenesis of the Striated Muscle Fibre, and the Growth of the Human Sartorius Muscle. Johns Hopkins Hospital Bulletin.

[B39] Merletti R. Parker P. (2004). Electromyography - Physiology, Engineering, and Noninvasive Applications. Hoboken, NJ: John Wiley& Sons.

[B40] Mesin L. (2013). Volume conductor models in surface electromyography: Computational techniques. Comput. Biol. Med. 43, 942–952. 10.1016/j.compbiomed.2013.02.002 23489655

[B41] Mesin L. Farina D. (2006). An analytical model for surface emg generation in volume conductors with smooth conductivity variations. IEEE Trans. Biomed. Eng. 53, 773–779. 10.1109/TBME.2006.872825 16686399

[B42] Miller G. L. Teng S.-H. Thurston W. Vavasis S. A. (1993). Automatic mesh partitioning, in Graph Theory and Sparse Matrix Computation (New York, NY: Springer), 57–84.

[B43] Mordhorst M. Heidlauf T. Röhrle O. (2015). Predicting electromyographic signals under realistic conditions using a multiscale chemo-electro-mechanical finite element model. Interface Focus 5, 1–11. 10.1098/rsfs.2014.0076 25844148 PMC4342944

[B44] Mordhorst M. Strecker T. Wirtz D. Heidlauf T. Röhrle O. (2017). POD-DEIM reduction of computational EMG models. J. Comput. Sci. 19, 86–96. 10.1016/j.jocs.2017.01.009

[B45] Negro F. Farina D. (2011). Decorrelation of cortical inputs and motoneuron output. J. Neurophysiol. 106, 2688–2697. 10.1152/jn.00336.2011 21795617

[B46] Pullan A. J. Cheng L. K. Buist M. L. (2005). Mathematically Modelling the Electrical Activity of the Heart: From Cell to Body Surface and Back Again. Singapore: World Scientific.

[B47] Qu Z. Garfinkel A. (1999). An advanced algorithm for solving partial differential equation in cardiac conduction. IEEE Trans. Biomed. Eng. 46, 1166–1168. 10.1109/10.784149 10493080

[B48] Rassier D. E. MacIntosh B. R. Herzog W. (1999). Length dependence of active force production in skeletal muscle. J. Appl. Physiol. 86, 1445–1457. 10.1152/jappl.1999.86.5.1445 10233103

[B49] Rau T. Krone M. Reina G. Ertl T. (2017). Challenges and opportunities using Software-Defined visualization in MegaMol, in Workshop on Visual Analytics, Information Visualization and Scientific Visualization (WVIS) in the 30th Conference on Graphics, Patterns and Images (SIBGRAPI'17), eds Ferreira N. Nonato L. G. Sadlo F. (Niterói).

[B50] Razumova M. V. Bukatina A. E. Campbell K. B. (1999). Stiffness-distortion sarcomere model for muscle simulation. J. Appl. Physiol. 87, 1861–1876. 10.1152/jappl.1999.87.5.1861 10562631

[B51] Reina G. Ertl T. (2005). Hardware-accelerated glyphs for mono-and dipoles in molecular dynamics visualization, in EuroVis, eds Brodlie K. Duke D. Joy K. (Leeds: CiteSeerX), 177–182.

[B52] Ríos E. Karhanek M. Ma J. González A. (1993). An allosteric model of the molecular interactions of excitation-contraction coupling in skeletal muscle. J. Gen. Physiol. 102, 449–481. 10.1085/jgp.102.3.449 8245819 PMC2229153

[B53] Röhrle O. Davidson J. B. Pullan A. J. (2008). Bridging scales: a three-dimensional electromechanical finite element model of skeletal muscle. SIAM J. Sci. Comput. 30, 2882–2904. 10.1137/070691504

[B54] Röhrle O. Davidson J. B. Pullan A. J. (2012). A physiologically based, multi-scale model of skeletal muscle structure and function. Front. Physiol. 3:358. 10.3389/fphys.2012.00358 22993509 PMC3440711

[B55] Röhrle O. Pullan A. J. (2007). Three-dimensional finite element modelling of muscle forces during mastication. J. Biomech. 40, 3363–3372. 10.1016/j.jbiomech.2007.05.011 17602693

[B56] Röhrle O. Sprenger M. Schmitt S. (2017). A two-muscle, continuum-mechanical forward simulation of the upper limb. Biomech. Model Mechanobiol. 16, 743–762. 10.1007/s10237-016-0850-x 27837360

[B57] Saad Y. Schultz M. H. (1986). Gmres: a generalized minimal residual algorithm for solving nonsymmetric linear systems. SIAM J. Sci. Statist. Comput. 7, 856–869. 10.1137/0907058

[B58] Schamberger S. Wierum J.-M. (2005). Partitioning finite element meshes using space-filling curves. Fut. Gener. Comput. Syst. 21, 759–766. 10.1016/j.future.2004.05.018

[B59] Shorten P. R. O'Callaghan P. Davidson J. B. Soboleva T. K. (2007). A mathematical model of fatigue in skeletal muscle force contraction. J. Muscle Res. Cell Motil. 28, 293–313. 10.1007/s10974-007-9125-6 18080210

[B60] Sundnes J. Lines G. T. Tveito A. (2005). An operator splitting method for solving the bidomain equations coupled to a volume conductor model for the torso. Math. Biosci. 194, 233–248. 10.1016/j.mbs.2005.01.001 15854678

[B61] Thomas L. H. (1949). Elliptic problems in linear difference equations over a network. Watson Scientific Computing Laboratory Report, Columbia University, New York, NY.

[B62] Valentin J. Sprenger M. Pfluger D. Rohrle O. (2018). Gradient-based optimization with b-splines on sparse grids for solving forward-dynamics simulations of three-dimensional, continuum-mechanical musculoskeletal system models. Int. J. Numer. Method Biomed. Eng. 34:e2965. 10.1002/cnm.2965 29427559

[B63] Wald I. Johnson G. Amstutz J. Brownlee C. Knoll A. Jeffers J.. (2017). OSPRay - a CPU ray tracing framework for scientific visualization. IEEE Trans. Visual. Comput. Graphics 23, 931–940. 10.1109/TVCG.2016.2599041 27875206

[B64] Wald I. Knoll A. Johnson G. P. Usher W. Pascucci V. Papka M. E. (2015). CPU ray tracing large particle data with balanced P-k-d trees, in 2015 IEEE Scientific Visualization Conference (Chicago: SciVis), 57–64.

[B65] Xia H. Wong K. Zhao X. (2012). A fully coupled model for electromechanics of the heart. Comput. Math. Methods Med. 2012:927279. 10.1155/2012/927279 23118801 PMC3480002

[B66] Zajac F. E. (1989). Muscle and tendon properties models scaling and application to biomechanics and motor. Crit. Rev. Biomed. Eng. 17, 359–411. 2676342

[B67] Zhou M. Sahni O. Devine K. D. Shephard M. S. Jansen K. E. (2010). Controlling unstructured mesh partitions for massively parallel simulations. SIAM J. Sci. Comput. 32, 3201–3227. 10.1137/090777323

